# Identifying silver ore sources for the earliest coins of Athens

**DOI:** 10.1007/s12520-024-02120-3

**Published:** 2025-01-25

**Authors:** Gillan Davis, Janne Blichert-Toft, Liesel Gentelli, Damian B. Gore, Kenneth A. Sheedy, Francis Albarède

**Affiliations:** 1 Faculty of Education and Arts, Australian Catholic University, Tenison Woods House, 8 Napier Street, North Sydney, NSW 2060 Australia; 2 Ecole Normale Supérieure de Lyon, CNRS, and Université de Lyon, Lyon, France; 3 School of Natural Sciences, Macquarie University, Sydney, Australia; 4 Department of History and Archaeology, Macquarie University, Sydney, Australia

**Keywords:** Lead isotope analysis, XRF, Athenian coins, Silver, *Wappenmünzen*, Owls

## Abstract

This study addresses longstanding questions concerning the ore sources used in the first series of coins of ancient Athens known as the *Wappenmϋnzen* (c.540-c.500 BCE) by combining comprehensive numismatic data on 22 coins (16 new and 6 legacy analyses) with lead isotope and surface elemental measurements (MC-ICP-MS and XRF). It finds usage of ores from Spain to Romania and Türkiye and frequent mixing. This upends current thinking based on a (mis)interpretation of historical sources which argues that the tyrant Peisistratos and his sons, who ruled Athens during the period, sourced most silver from the districts of Mt Pangaion and Strymon River in northern Greece and that silver did not flow from the western Mediterranean into their coinage. The data suggest that domestic ‘Lavrion’ mines of Athens did not contribute to the ore stock of the *Wappenmϋnzen* until the subsequent production of the ‘owl’ series when it was also used in some *Wappenmϋnzen* fractions and show that there is no correlation between coin types and ore sources. Elemental compositions nuance our understanding of the coins, but do not shed light on provenance. Together, these new findings force a reappraisal of numismatic and historical perceptions of the period of the Athenian tyranny in the lead up to democracy, not least because the multiple silver sources point to trading relationships with a greater variety of regions than previously contemplated.

**Supplementary Information:**

The online version contains supplementary material available at 10.1007/s12520-024-02120-3.

## Introduction

Sources of silver used by the ancient Athenians to mint their first coins in the late Archaic period (c.540–479 BCE) have long been a subject of debate. The discussion has centred on mining districts mentioned anecdotally by historical authors, coinages and observed ancient workings (Gale et al. 1980; Gentner et al. 1978; Kraay and Emeleus 1962). The scientific contribution has largely been informed by lead isotopic and chemical analyses carried out over 40 years ago on coins in the Asyut hoard (*IGCH* 1644 c.475 BCE) by Gale et al*.* (1980, revised Stos-Gale and Davis 2020), but the fact that so many of these coins are from northern Greek mints skews the data. This paper provides fresh understandings based on new lead isotope and X-ray fluorescence (XRF) elemental analyses combined with legacy data comprising 22 coins (this study: 16 coins from the Athens Numismatic Museum and Gale et al.: 6 coins from the British Museum, excluding a seventh coin which is unidentifiable) from the earliest series of coins minted by the Athenians known as the *Wappenmϋnzen* (literally ‘heraldic coins’ so named for their changing types once erroneously thought to be blazons of noble families, cf. Sheedy et al. 2009). The elemental and isotopic data are combined with numismatic information on the coins and their types (Sheedy and Davis forthcoming). The key aim of the study is to determine the major silver ore sources accessed by the Athenians to produce the *Wappenmϋnzen.* The surprising answer has important historical and numismatic implications.

Athenian coinage was instigated by the Peisistratid tyrants who ruled Athens from 546 to 510 BCE. The first coinage series is known as *Wappenmϋnzen* and is characterised by multiple changing types (13 silver types identified in Sheedy and Davis, forthcoming). Of these, the most important were the ‘horse’, ‘gorgon’ and ‘wheel’, each of which had their own varieties. They were followed by the long-lasting series for which Athens’ coinage became best known – the ‘owl’ – which depicted the helmeted head of Athena on the obverse and an owl on the reverse along with an olive sprig, another symbol of the goddess, and the abbreviated ethnic – ΑΘΕ (meaning: coin of the Athenians). The change to the owl as the sole type reflected a desire to have an instantly recognisably Athenian currency like other leading Greek trading states such as Korinth and Aigina (Kraay 1956). This decision has been linked with the exploitation of silver-rich mineral veins in the mining district at Lavrion (Laurion) in south-east Attica (Davis 2014a), since the owl type was minted principally in large quantities of high-value tetradrachms intended for export, in contrast to the small obols and didrachms of the *Wappenmϋnzen* which mainly circulated locally (Kraay 1956).

Literary evidence from long after the events described above took place implies, but does not prove, that the Peisistratids derived the silver for the *Wappenmϋnzen* from the districts of Mt Pangaion and Strymon River in northern Greece (Hdt. 1.64.1; *Ath. Pol*. 15.2) followed by exploitation of Lavrion for the owls (Hdt. 7.144). This has been widely accepted since the theory was first popularised by Seltman (1924; cf. van Wees 2013; Sears 2013, 2015) notwithstanding serious doubts of other scholars, notably Lavelle (1993; cf. Archibald 2013 and Davis 2014a). Lead isotope analysis of *Hacksilber* (chopped silver) from Tel Miqne-Ekron in the southern Levant shows that one of its sources was Lavrion in the seventh century BCE (Gentelli et al. 2021; Stos-Gale 2001) and there might be an expectation that exploitation of Lavrion continued into the sixth century BCE. Against this, there is virtually no evidence of silver use in Attica prior to the second half of the sixth century (Davis 2012 contra Rhodes 1975; Kroll 1981). Gale et al. (1980) influentially assumed that silver from the western Mediterranean was ‘virtually unavailable’ to the Greeks after the battle of Alalia in 540 BCE won by the Carthaginians. This was always a somewhat counterintuitive conclusion since their own lead isotope analyses of seven *Wappenmϋnzen* coins claimed to represent diverse sources ranging from Spain to Iran with only one fractional (small) coin late in the sequence of types from Lavrion, and all 14 owls exclusively from Lavrion ores (Gale et al. 1980; re-examined by Stos-Gale and Davis 2020).

Some gorgoneion *Wappenmϋnzen* were minted as tetradrachms with an image on the reverse of a lioness rather than the unadorned diagonally divided reverse punch used hitherto (Table 1). Kraay (1956) in a seminal article argued that these were the last phase of the *Wappenmϋnzen* with their increased size connected to a drop in the value of silver late in the sixth century BCE caused by increased production including perhaps from Lavrion. Van Alfen (2012) explicitly linked the choice of the larger denomination to exploitation and export of Lavrion silver. This causal connection is tested in this paper since it has been shown that small-denomination *Wappenmϋnzen* continued to be minted without a developed reverse after the introduction of the owls (Kroll 1981 for some gorgons and Davis 2014b demonstrating the more substantial volume of wheels).

**Table 1:**
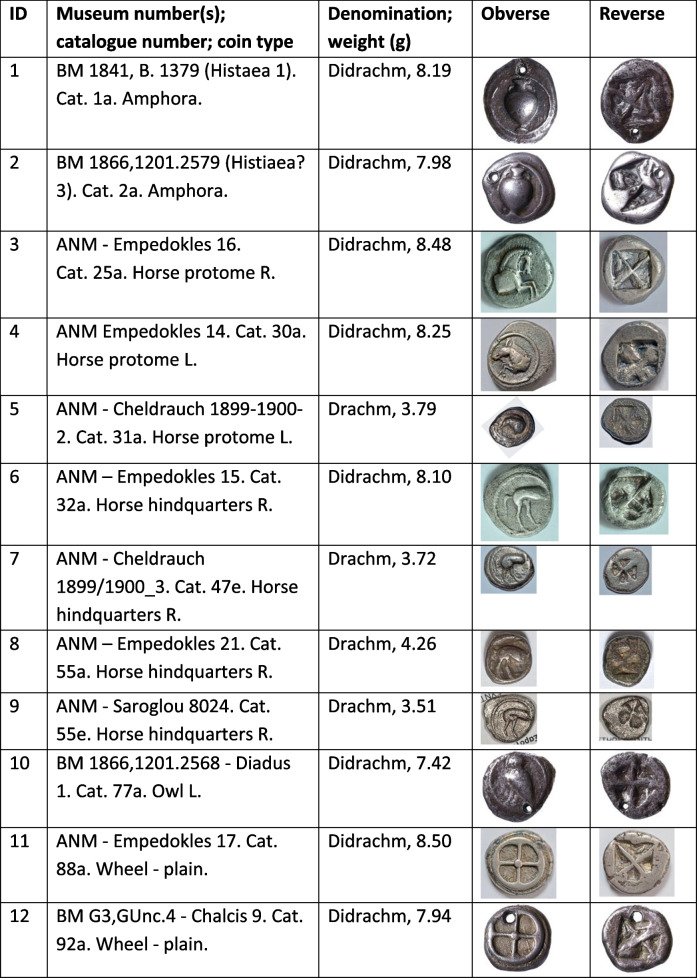

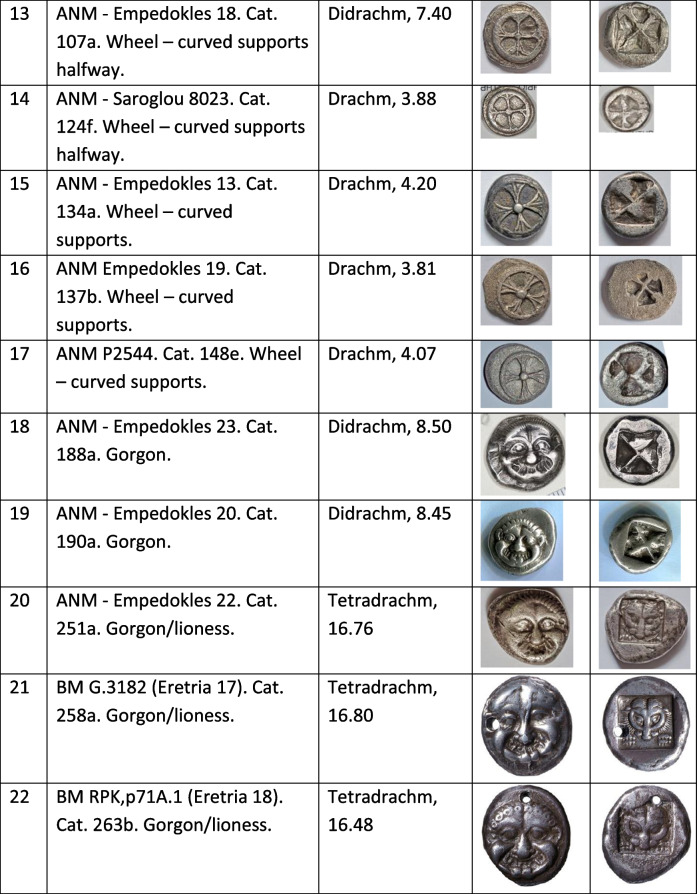
Coins analysed in this study. Coin museum and catalogue numbers are from Sheedy and Davis (forthcoming); obverse and reverse images not to scale. Photos by the authors

Our understanding of Greek ore sources has greatly expanded since the 1980s. Reasons include use of more sensitive and accurate analytical equipment for high-precision Pb isotope measurements (Blichert‐Toft et al. 2003); wider sampling of Aegean and northern Greek mines (Vaxevanopoulos et al. 2022), Spanish mines (Milot et al. 2021a, b) Balkan mines (Westner et al. 2023), and Turkish mines (Sayre et al. 2001); better understanding of ore formation (Albarède et al. 2012) and the significance of their geological settings (Blichert-Toft et al*.* 2016) in Pb isotope space; narrowing of possible ore sources through silver isotope analyses (Albarède et al. 2021; Milot et al. 2021a, b; Vaxevanopoulos et al. 2022); and new statistical approaches (Albarède et al. 2020; Albarède et al. 2024a, b).

## Materials and methods

### Description

We sampled 16 *Wappenmϋnzen* coins from the Athens Numismatic Museum. We merged our data with legacy data which consist of six *Wappenmϋnzen* from the British Museum (Gale et al. 1980). These latter data were used with reservation since they were produced using thermal-ionisation mass spectrometry (TIMS) which was state-of-the-art at the time but does not control analytical mass bias as efficiently as multiple-collector inductively coupled plasma mass spectrometry (MC-ICP-MS), which is the method most commonly used for Pb isotope analyses today, including in this study. However, because the algorithm used in the present work handles mass-dependent isotope fractionation to some extent, the legacy data can be reconsidered. To ignore these data would not only be unscholarly but would also be to lose much valuable information on the *Wappenmϋnzen*. We therefore keep the legacy data in our database but interpret them with caution. The coins and their images are listed in Table 1. The sampled coins represent 7% of 318 known examples excluding fractions (Sheedy and Davis forthcoming).

### Lead isotopic analyses

Coins sampled at the Athens Numismatic Museum were analysed for their Pb isotopic compositions at the Laboratoire de Géologie of the Ecole Normale Supérieure de Lyon (ENS Lyon) using a virtually non-destructive technique whereby only a few micrograms of material were removed from each coin. The methods and analytical precision and accuracy are detailed in Milot et al. (2021a, b). In summary, the coin was rolled on its edge for c.90 s onto a strip of chromatographic paper impregnated with a solution of H_2_O_2_, NH_4_OH, and H_2_O in the proportions of 1:1:1. The strips were then air-dried under an infrared lamp, placed into clean 10 mL centrifuge tubes wrapped in multiple layers of film to avoid contamination and carefully transported from the sampling site to the clean laboratory at ENS Lyon for Pb separation and isotopic analysis. In the clean laboratory, 10 mL double-distilled 1 M HBr, for which Pb has strong affinity, were added to the tubes submerging the strips which were left to leach at ambient temperature for 24 h. The HBr containing the Pb leached from the strips was transferred to a clean Savillex (PFA) beaker and evaporated to dryness at c.130˚C on a hot plate. The HBr-leached strips were then submerged in 10 mL distilled 0.5 M HNO_3_ and left to leach further at ambient temperature for another 24 h. The HNO_3_ was transferred to the same beakers containing the now dried-down HBr solutions and evaporated to dryness under the same conditions. Lead from the leachates was then separated and purified by anion-exchange chromatography (on 0.5 mL AG1-X8, 100–200 mesh micro-columns) using double-distilled 1 M HBr to elute the sample matrix and distilled 6 M HCl to collect the Pb. The Pb isotopic compositions were measured by MC-ICP-MS (Nu Plasma 500 HR) using added Tl to correct for instrumental mass bias and applying sample-standard bracketing for accuracy of the measured samples relative to the NIST 981 Pb standard using the triple-spike TIMS values of Eisele et al. (2003).

### Elemental composition

Elemental analyses were conducted on-site in the Athens Numismatic Museum and in the British Museum using a PANalytical Epsilon3 benchtop XRF with 50 kV Rh anode tube. Coins were measured in air on both sides for 100 s with a spinner at 1 Hz. Standardless quantification in automatic mode provided automated qualitative spectrum analyses. The calibrations used for the fundamental parameter matrix model supplied by the instrument manufacturer were refined and analytical inaccuracy constrained with 12 matrix-matched certified reference materials from MBH Analytical Ltd. The automated deconvolution of every spectrum was checked manually. The same designated Ag coin was analysed twice daily to help correct for instrumental drift. Generally, elements with concentrations of < 0.1 wt% have relative errors of 10–100%, and elements present at > 1 wt% have relative errors of < 10%. To correct for differences between surface and bulk compositions, a simple mathematical correction was adopted (Gore and Davis 2016).

### Statistical method

The Lyon Pb isotope database comprises isotopic data for 7,000 galena ore samples sourced from regions across the Mediterranean and Europe. This database includes essential information such as sample names, regions of origin, geographic coordinates, Pb isotope ratios, model ages, mu and kappa values, and references to the original studies. Initially established by Delile et al. (2014), the database undergoes continual updates. However, a significant portion of the data originates from books, PhD dissertations, and unpublished reports, where detailed data protocols are sometimes lacking. Consistency between this database and the Bochum GlobalID database has been demonstrated (Klein et al. 2022). The Lyon Pb isotope database is available upon request.

The algorithm, which accounts for mass-dependent isotope fractionation and applies a chi-squared test to the distance distribution, is detailed by Albarède et al. (2024a), particularly in their Fig. 2. In summary, the data are centered and normalized according to analytical errors with a complete correlation structure, and the distance between the mass-fractionation lines of a given coin and each ore sample is calculated. The squared distance *d* between these lines follows a chi-squared distribution with two degrees of freedom. To minimize the inclusion of mixed bullion sources, we reduced the critical chi-squared value from 5.99 to 1, thereby discarding more than half of the potential matches. This method marks a significant advancement over traditional Euclidean distance methods initiated by Sayre et al*.* (1975) and subsequently refined by numerous authors. Our approach is thus driven by the data rather than the coin, recognizing that the ore used to produce the coin is no longer in the mine and a one-to-one match is impossible.

Accurately pinpointing ore source(s) for individual coins as opposed to quantifying the ore contributions for many coins may be problematic if the ore source does not exist in the database or, possibly, a source is discounted as being unlikely when it did actually contribute ore. This could prove to be the case with hitherto unsuspected ancient mining districts such as in the south of France and North Africa which is why we present more than one of the top hits in our results. Untangling mixed sources is another problem discussed at length in Albarède et al. (2024a and b) in which a ‘Sardinia mix’ was identified for some western silver, notably from southern Sardinia, Sierra Morena, and southern France, that was evidently circulating in the Mediterranean prior to being struck into coins in various places including Athens.

## Results

**Fig. 1:**
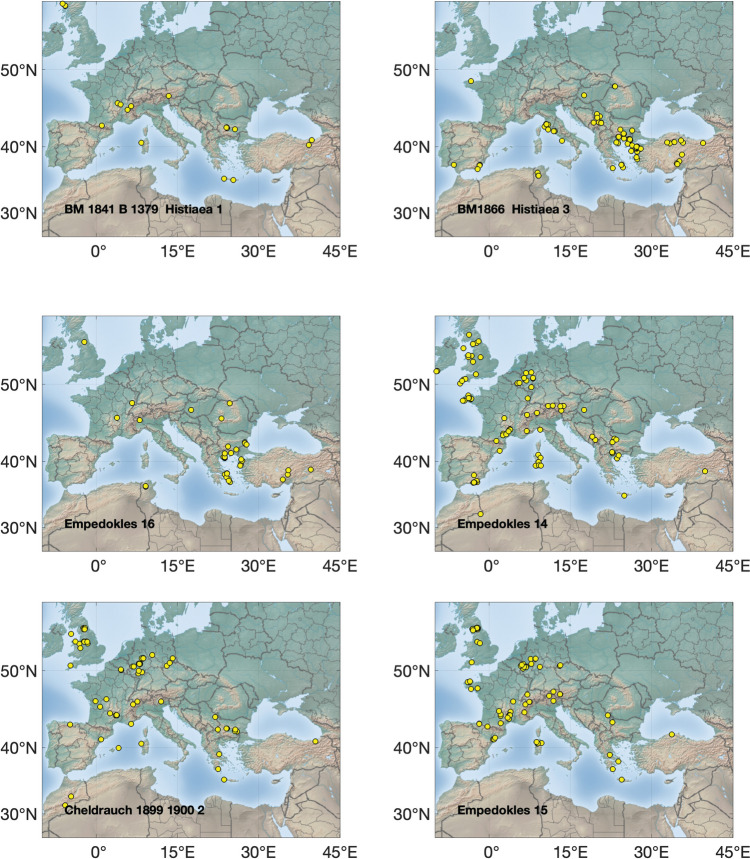
Coins 1–6. Matches (chi-squared < 1) obtained for the analysed coins by the procedure described in Albarède et al. (2024a). Mass-dependent isotope fractionation is taken into account by the algorithm

**Fig. 2:**
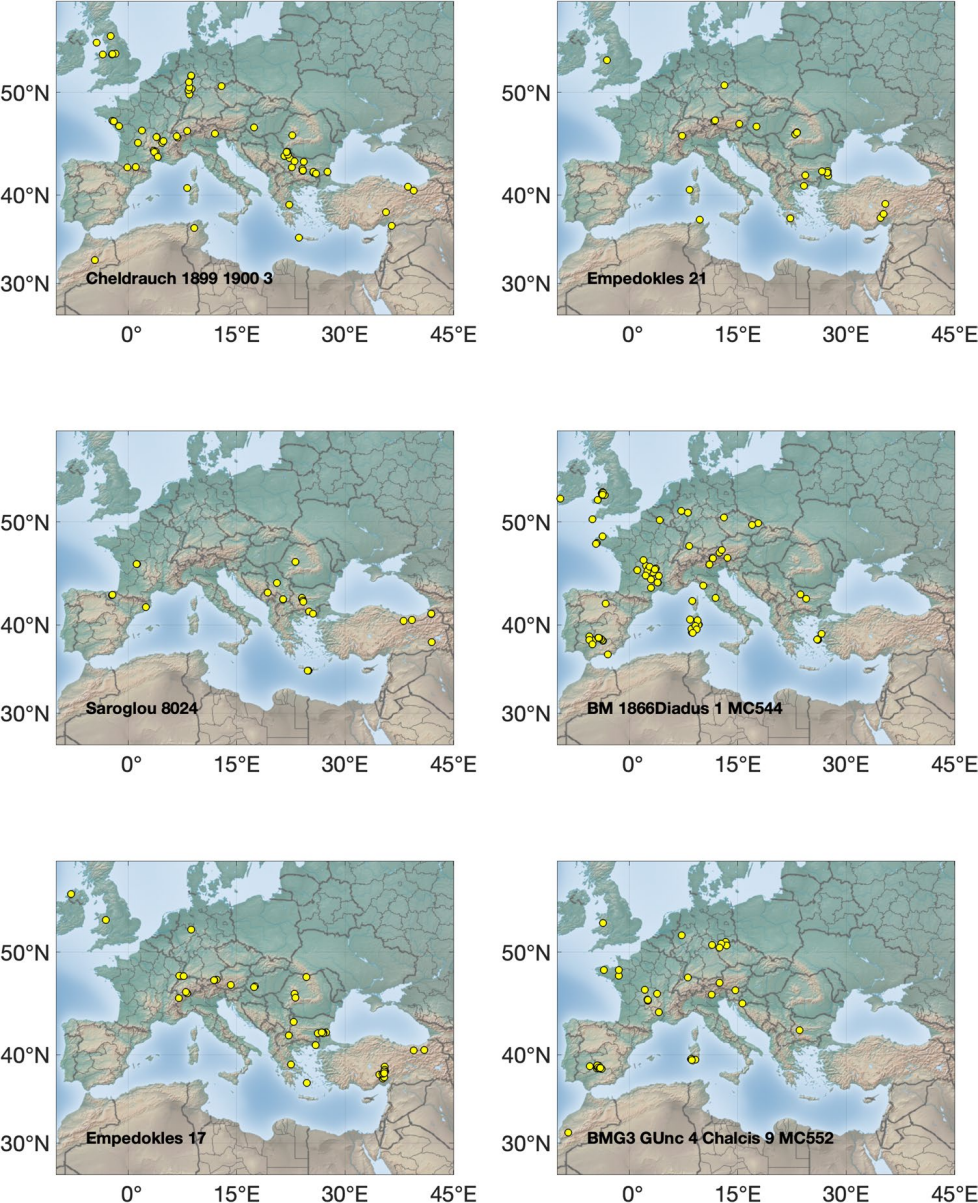
Coins 7–12

**Fig. 3:**
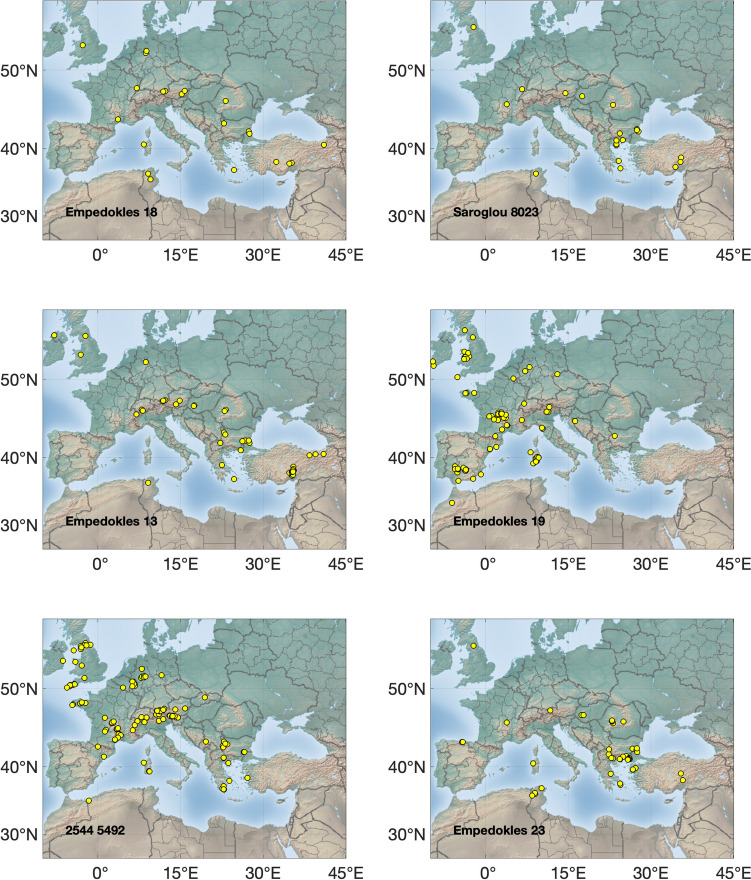
Coins 13–18

**Fig. 4:**
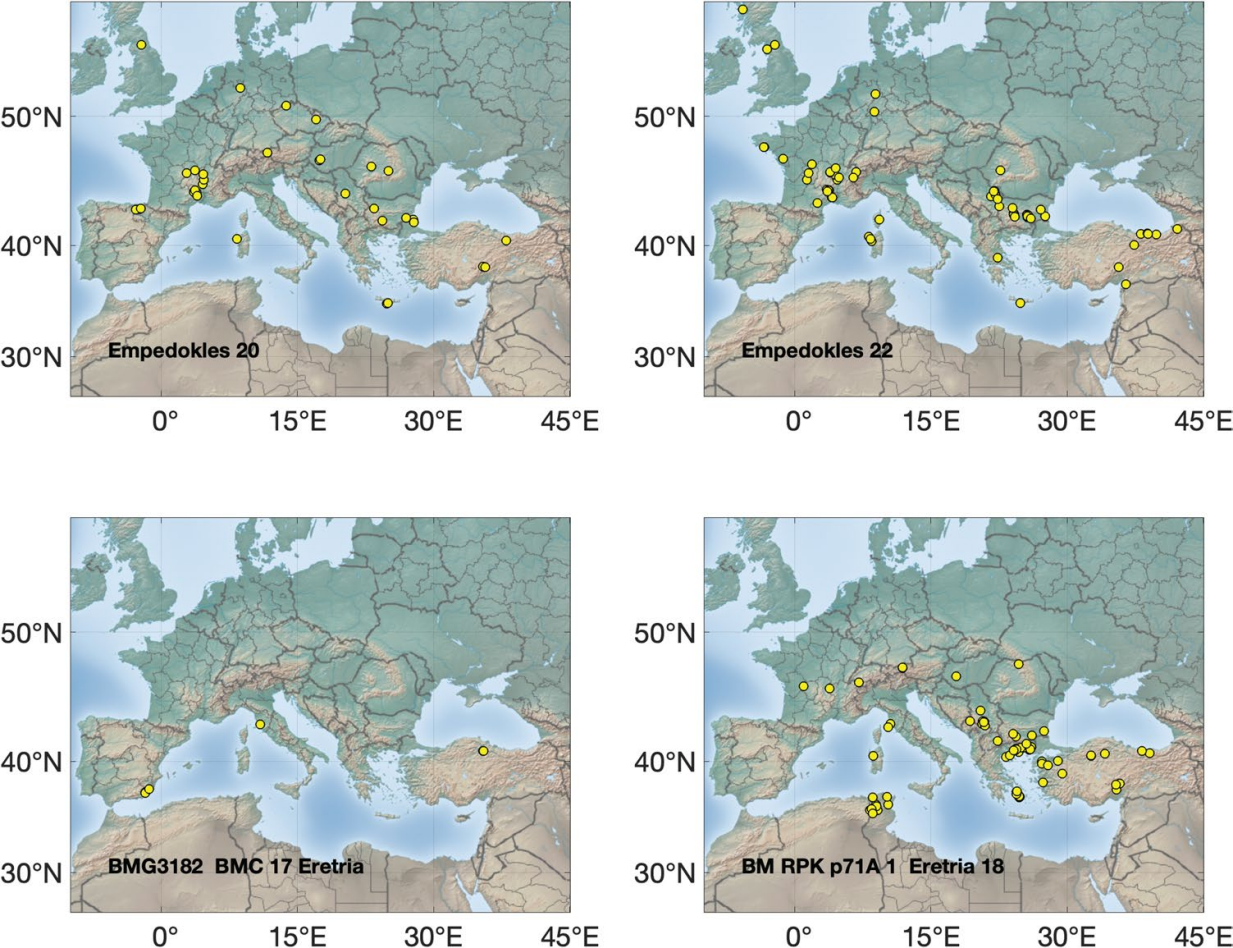
Coins 19–22

**Fig. 5:**
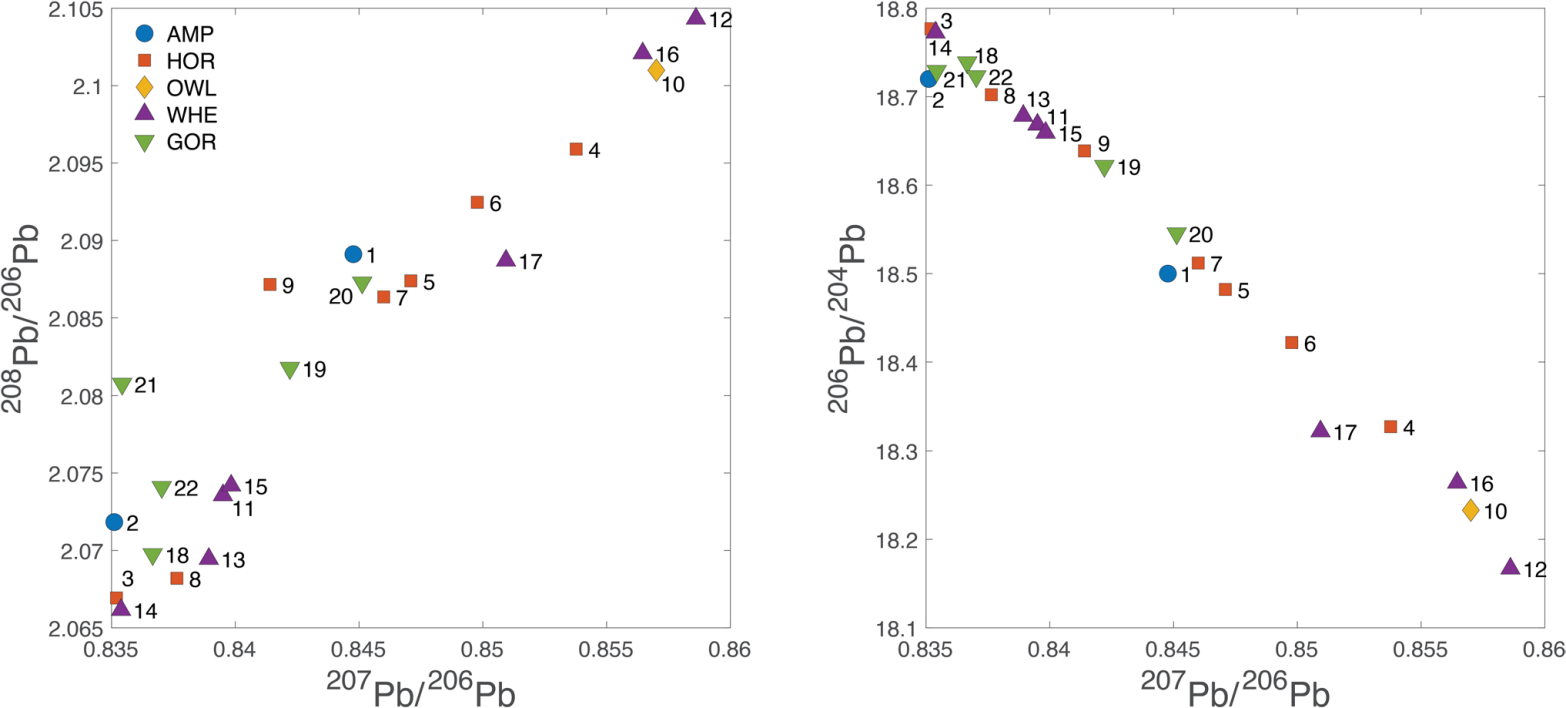
2D plots of the coins with the coin types (amphora, horse, etc.) colour coded as per Table 2

**Fig. 6:**
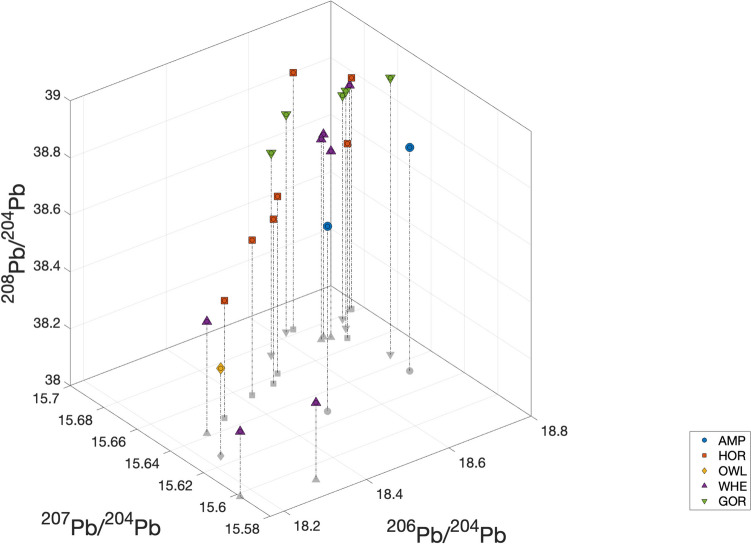
Comparison of the 3D data representation with the 2D data representations of Fig. 5. The 3D plot shows that, unlike the 2D plot of ^206^Pb/^204^Pb vs ^207^Pb/^206^Pb, there are no subgroups defining a linear array (mixing)

The Pb isotopic data are listed in the supplementary materials ESM1, ESM2 and ESM3 and presented graphically in several ways: as ‘hit’ maps for each coin (Figs. 1, 2, 3 and 4) and as conventional 2D and 3D plots (Figs. 5 and 6). Conclusions about the data sources drawn from the hit maps are summarized in Table 2. Major and trace element abundances are listed in Table 3. The full data set is provided in the supplementary materials ESM1, ESM2 and ESM3. Full size, coloured versions of the hit map for each coin are also included in the supplementary materials ESM1, ESM2 and ESM3 where black dots signify the highest probability through to yellow for the lowest probability.

**Table 2:**
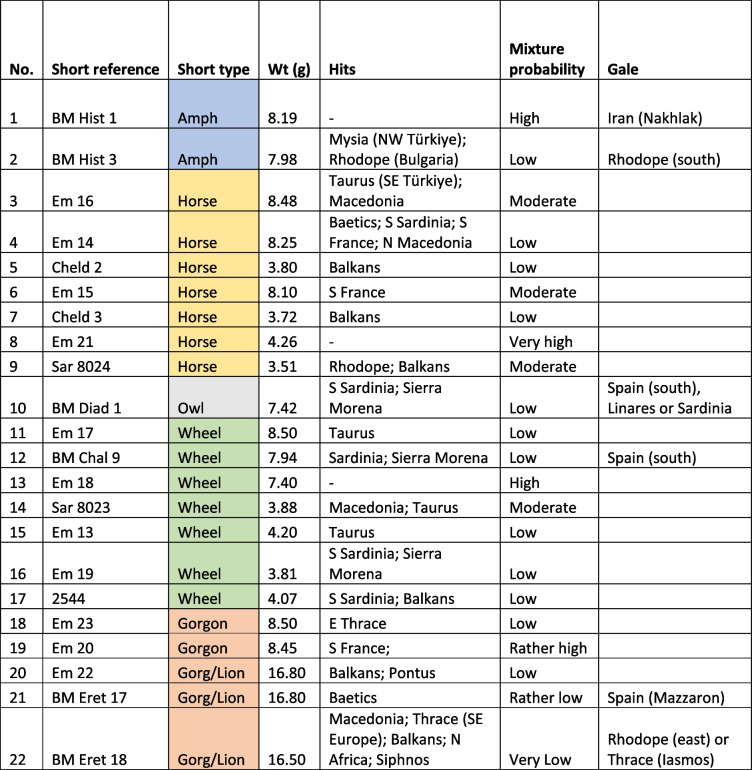
Summary of the results and interpretations of coins 1–22 (Figs 1-4). The results are summarized and colour-coded by type. Coins without hits have a very high probability of mixture of three sources or more. The level of certainty for mixing is indicated from low to very high

**Table 3:**
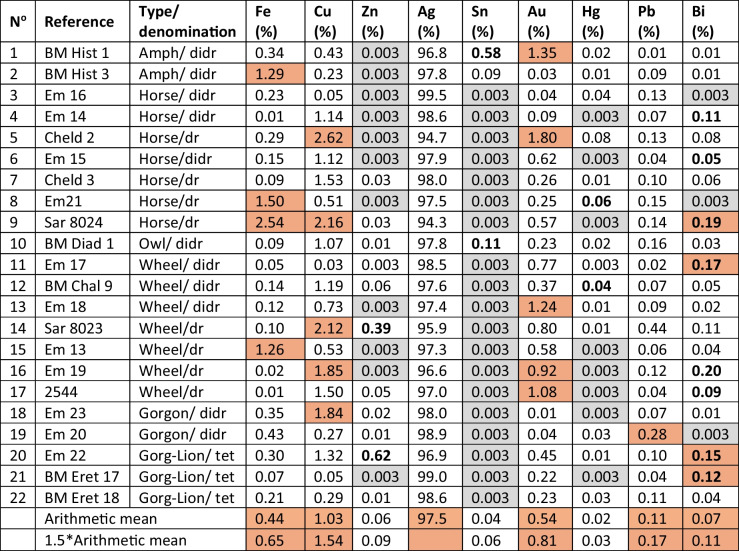
Elemental concentrations of the coins analysed in this study

### Hit maps

The hit maps for the coins are accompanied by a suggestion in each case of the most likely source(s) (see supplementary materials ESM1, ESM2 and ESM3). Coins are identified by their short title. Where matches overlap, the results have been jittered to display them more clearly.

### 2D and 3D plots

The data represented in 2D plots (Fig. 5) show a lack of correlation between coin types and ore sources indicating that coins were struck from whatever sources were at hand throughout the series. Note that ore fields commonly shown on such plots are not added since these are misleading as we have demonstrated previously (Albarède et al. 2020).

### Elemental analyses

As mentioned above, the elemental compositions of the 22 coins analysed in this study are given in Table 3. Vanadium, Cr and Ni analyses have been omitted because all of their concentrations were < 0.01%. Values below the limits of quantification were substituted by n/3 (typically rounded to 0.003% and the cells shaded grey) in order to calculate arithmetic means. Brown shading shows coins which exceed 1.5* the arithmetic mean concentration of an element, except for Zn, Sn and Hg due to large numbers of values less than the limit of quantification of 0.01% where the relevant coins are in bold. Numbers in bold under Bi indicate where its concentration is > Pb.

## Discussion

Judgement is required to determine whether a hit is meaningful. In general, hits for ores with no archaeological or literary evidence of ancient Ag production have been disregarded, noting that it is the Pb being analysed, not the Ag. For instance, the Peloponnese Peninsula had lead mines but no viable silver. Likewise, hits from places too far away to have contributed ore, such as central and northern Europe and the UK, are not likely sources either. Isolated hits are also not sufficient to be considered a source. However, the *absence* of strong hits is probably indicative of mixtures and hence informative.

The results show that the Athenians used a wide and unexpected variety of ore sources for the minting of their first series of coins, ranging from Spain in the west to the south of France through to Türkiye in the east and the Rhodope Mountains in the north. This reinforces the idea that minting was opportunistic (Stos-Gale and Davis 2020). The state minted whatever supplies of silver came to hand and frequently these were mixed either at the mint in Athens or in the wash of commerce around the Mediterranean mediated by the Athenians and probably others such as the Euboians and Aiginetans. The silver itself knew no bounds since it was a universal medium of exchange, but it had to be transported by traders to Athens and they were evidently not prevented from doing so either by the Phoenicians or the Persians as Gale et al. (1980) had supposed. This is supported by the ancient literature. Kolaios the Samian had sailed to Tartessos in Spain in the seventh century BCE and brought back the immense sum of 60 talents and Sostratos the Aiginetan even more (Hdt. 4.152); others would have been inspired by the tales. In the early sixth century BCE Solon the Lawgiver of Athens was renowned for his decades of travel including to Egypt and Lydia in Türkiye (Hdt. 1.29–33), while Alkmeon, the founder of the fortune of the Athenian Alkmeonid clan was covered in gold by King Kroisos of Lydia (Hdt. 6.125). In the second half of the sixth century, Peisistratos’ establishment of a base at Rhaikelos and Mount Pangeion in the northern Aegean has already been noted (*Ath. Pol*. 15.2) and although he was not directly mining silver there, it arguably gave him access to trade from the northern hinterland up to the Rhodope Mountains and even Romania and around the coast of the northern Aegean. Davies (2013) has argued that Peisistratos established ‘corridors’ of outreach linking Athens with the north Aegean. Peisistratus had close connections with the Cyclades (Hdt. 1.64.2 for his aid to the tyrant Lygdamis in his conquest of Naxos and purification of Delos), and he stepped up activity in the east. He established a colony at Sigeion in the Troad at the mouth of the Hellespont (Hdt. 5.94) and acquiesced in a leading aristocrat Miltiades establishing a principality on the Khersonese in the Dardanelles (Hdt. 6.34). Furthermore, the Athenians had longstanding close connections with the Ionians, whom they considered their kin, especially Peisistratos who claimed shared ancestry with Neleus, the founder of Miletos on the coast of Asia Minor (Hdt. 5.65.3) and Melanthos who was the mythical founder of at least eight other Ionian towns (see Davies, 2013 for sources). It is therefore understandable to see the significant contribution of silver ore from Turkish mines. The Taurus identification is an outcome of our group’s recently published algorithm (Albarède et al. 2024a) which deals with isotope fractionation (whether natural or instrumental) and introduces a measure of statistical 'distance' between data in the 3D space of Pb isotope ratios. Importantly, the near absence of silver ore from the district in northern Greece where the Peisistratids established themselves in exile for ten years finally proves that the wealth (*chrēmata* in Greek) they derived specifically from there was not from their own silver mining.

The absence of silver from Lavrion for the gorgons minted in the large tetradrachm denomination at the end of the minting of the larger denomination *Wappenmϋnzen* series is unexpected. Although the size of the sample (N = 3/33 known examples) cannot make this finding definitive, whatever the reason for their minting, it should not be linked to exploitation of domestic ores, which seems to have come later than their production. This is not from misinterpretation of the data. When a coin contains even a small proportion of Lavrion ore, the hits clearly demonstrate that fact because we have more data by far on Lavrion ores than on ores from anywhere else. To consolidate this assertion, we observe the use of Lavrion ore *both* in the subsequent owl series *and* in a high proportion of the later issued fractional *Wappenmϋnzen* coins, which co-existed alongside the owls (Davis et al. 2020).

Multiple hits in the Bulgarian coastal volcanic province could suggest the possibility of a forgery as the region has no silver mines and the ancients did not know how to extract silver from the abundant copper ores, the technology to do so only being developed in the fifteenth century AD using cyanide and amalgamation extraction methods (L’Héritier and Téreygeol 2010). However, this possibility must be discounted on numismatic grounds. The contention is well demonstrated for coins 8 and 9 which share the same dies along with three other known examples. The former has strong hits in Bulgaria while the latter is most likely from Thrace. Furthermore, none of the coins are sufficiently rare or valuable to warrant forging. It is more likely that the Bulgarian hits represent an unidentified mining region.

There is no clear correlation between coin type and ore source. The two amphoras are among the earliest in the *Wappenmϋnzen* sequence. They are different varieties of the type of which only six are known in didrachms. Coin 1 was purchased in Athens in 1832 and coin 2 was acquired by the British Museum in 1866. Their ore sources are both in Türkiye, but the former is probably from Pontus on the southeastern shore of the Black Sea and the latter from Mysia on Propontis or possibly the Rhodope Mountains. An even clearer demonstration that the same ore was not used to strike coins from identical dies comes from coins 8 and 9 (discussed above). Both are drachms with the obverse design of the hindquarters of a horse standing to the right and a simple *chi* (X) reverse, but they have two distinct sources. Similarly, the three gorgon tetradrachms mentioned earlier, all with impeccable numismatic credentials (coins 20, 21 & 22), have distinct ore sources.

Analysis of the elemental data reveals some interesting findings. Cells in Table 3 shaded in brown have concentrations of elements higher than 1.5* the arithmetic mean. It is clear that there is no correlation between composition and provenance. Even coins that plot very closely together, such as coins 11 & 15 and 10 & 16, do not have similar compositions or even key diagnostic elemental concentrations in common. However, only one coin (coin 2, an amphora) has the combination of low diagnostic elements common to Lavrion ores and its ore provenance is Mysia. This supports the finding that Lavrion ores were not used for the 22 sampled *Wappenmϋnzen*. The coins were minted to a consistent purity of Ag averaging 97.5% throughout the series. Only coin 9 has three elements significantly higher than the mean. Four coins have high levels of Fe, but this is likely due to circumstances of deposition as Fe is abundantly present in all soils and readily migrates into coin surfaces (Gore and Davis 2016). Four coins have low Cu but five have Cu > 1.54% with three more at around the same level. This Cu must have been added (Davis et al. 2020). Zinc is in two coins with significantly high concentrations (coins 14 & 20). Tin is also in two coins with high concentrations (especially coins 1 & 10), but it is difficult to read too much into this as Sn is difficult to quantify at these concentrations with the XRF used (Gore and Davis 2016). Gold is low for four coins and high for five. Mercury is low in all coins though two are slightly elevated. Lead is low in 12 coins, sometimes extremely low, notably coin 1 which is correspondingly high in Au and Sn. This coin is among the earliest in the series (amphora) and it is possible that it is not from cupellated Ag. It is also interesting to note that Bi concentrations exceed those of Pb for seven coins. This is unusual as a wider study of early Athenian coins (*Wappenmϋnzen* and owls) showed that while Bi can only derive from initial concentrations, it usually follows Pb as it boils off at a lower temperature (1564° C compared with 1749° C; L'Héritier et al. 2015; Davis et al. 2020).

## Conclusions

The key finding of this study is that the Athenians used a wide variety of ore sources to mint their earliest coin series. These ranged from Spain to the south of France as far as Türkiye, and into northern Greece and the Balkan Peninsula and as far north as Romania. Much of it was mixed. This points to undocumented trading relationships and a much more interconnected world than hitherto thought, though it does not mean that all or even most of the trade was physically carried out by Athenians.

It is in this context that the stories about the activities of the Peisistratids in the northern Aegean need to be reconsidered. They clearly did not mine silver themselves as the silver for their coinage was not extracted from mines near their base. However, they may well have used their position in northern Greece to connect into the routes along which silver was traded, bearing in mind that the volume of production of the larger denomination *Wappenmϋnzen* was relatively small. Davies (2013) argued that the Peisistratids also made efforts to expand their influence into the Dardanelles and this seems to have had a by-product of connecting Athens into the trade in silver mined in Türkiye. The Taurus region, which had produced silver for millennia before a likely cessation in the 9th c. BCE (Weeden 2010), was evidently producing silver again as were the mines at Mysia in the former Lydian kingdom, or perhaps it was just a case of some silver from these regions being recycled back into the Aegean.

On the basis of the samples analysed, the Peisistratids did not derive silver from domestic sources in Attica when the gorgon/lioness tetradrachms were struck. The exploitation of the Lavrion mines should be connected with the introduction of the subsequent owl series and for its use in the continued production of fractional *Wappenmϋnzen*. There was no correlation between the source of the ore and the type or variety of type used to strike some of it into coinage.

Compositional analyses provide little assistance for provenance studies except that gold is a useful marker of non-Lavrion ores. The consistently high purity of silver is noted. Copper was added probably more to harden the coins than to make a profit through diluting the silver content. The presence of impurities indicates a lack of refinement in cupellation.

## Supplementary Information

Below is the link to the electronic supplementary material.

Supplementary file1 (XLSX 14.0 KB)

Supplementary file2 (ZIP 8.20 MB)

Supplementary file3 (ZIP 65.3 KB)

## Data Availability

Data are provided within the manuscript or supplementary information files.
