# Supplementary figures and images for: Identifying silver ore sources for the earliest coins of Athens
Source: Archaeol Anthropol Sci. 2025 Jan 25;17(2):45. doi: 10.1007/s12520-024-02120-3 (PMC11762618; doi:10.1007/s12520-024-02120-3)

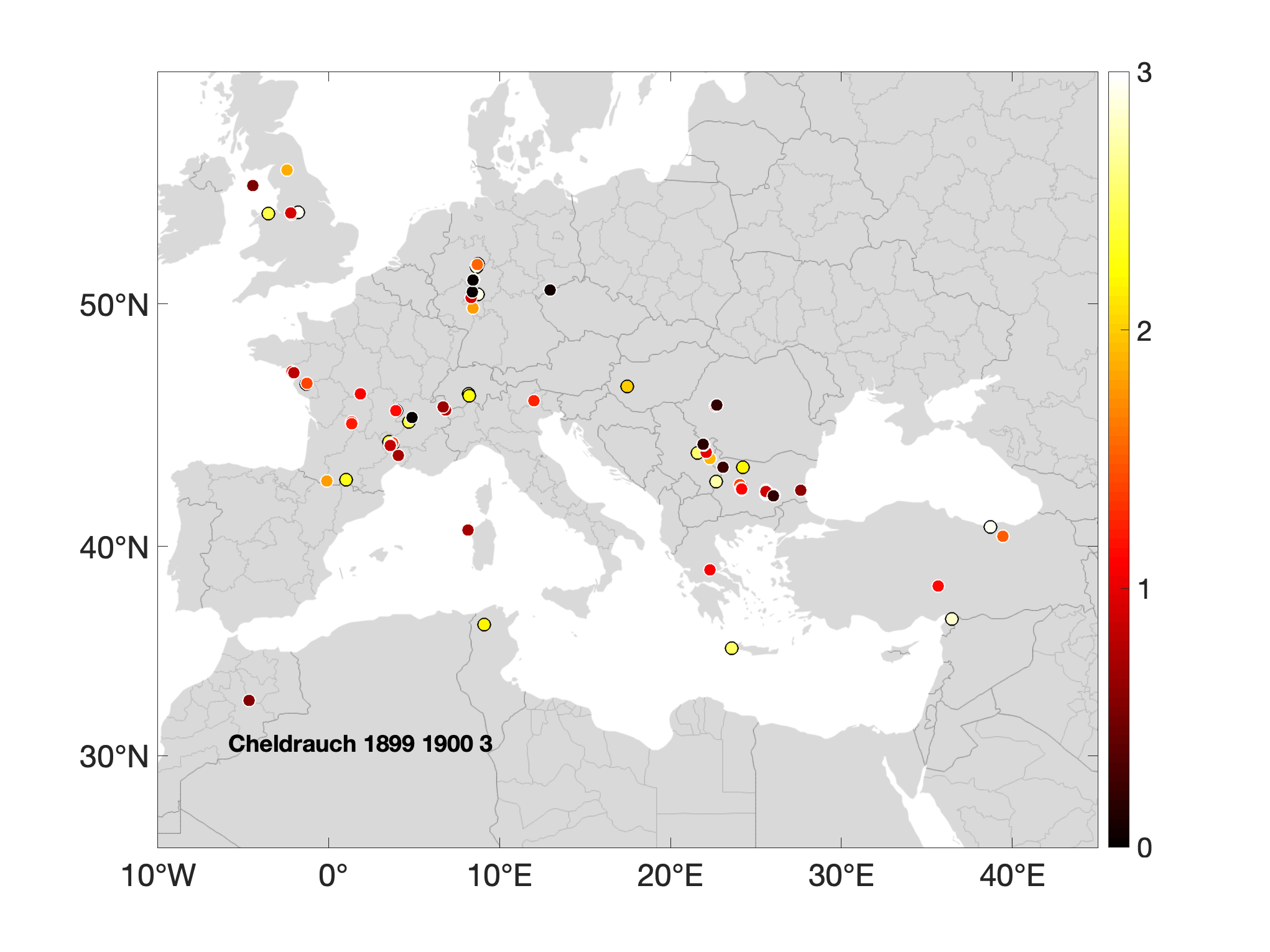

Supplement: Supplementary file 2 — Supplementary file2 (ZIP 8.20 MB) [file 12520_2024_2120_MOESM2_ESM.zip › png/Cheldrauch 1899 1900 3_map_jittered.png]

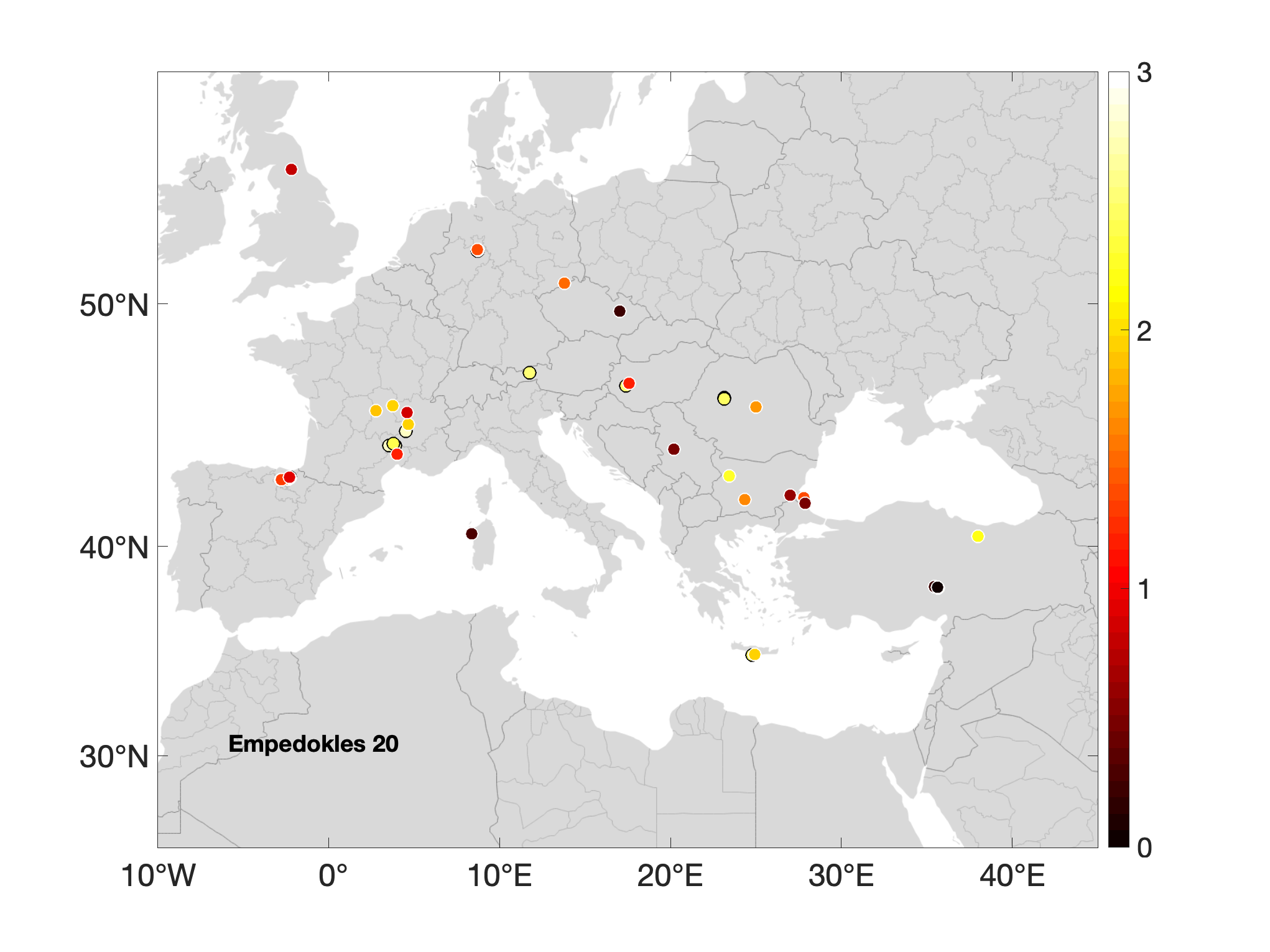

Supplement: Supplementary file 2 — Supplementary file2 (ZIP 8.20 MB) [file 12520_2024_2120_MOESM2_ESM.zip › png/Empedokles 20_map_jittered.png]

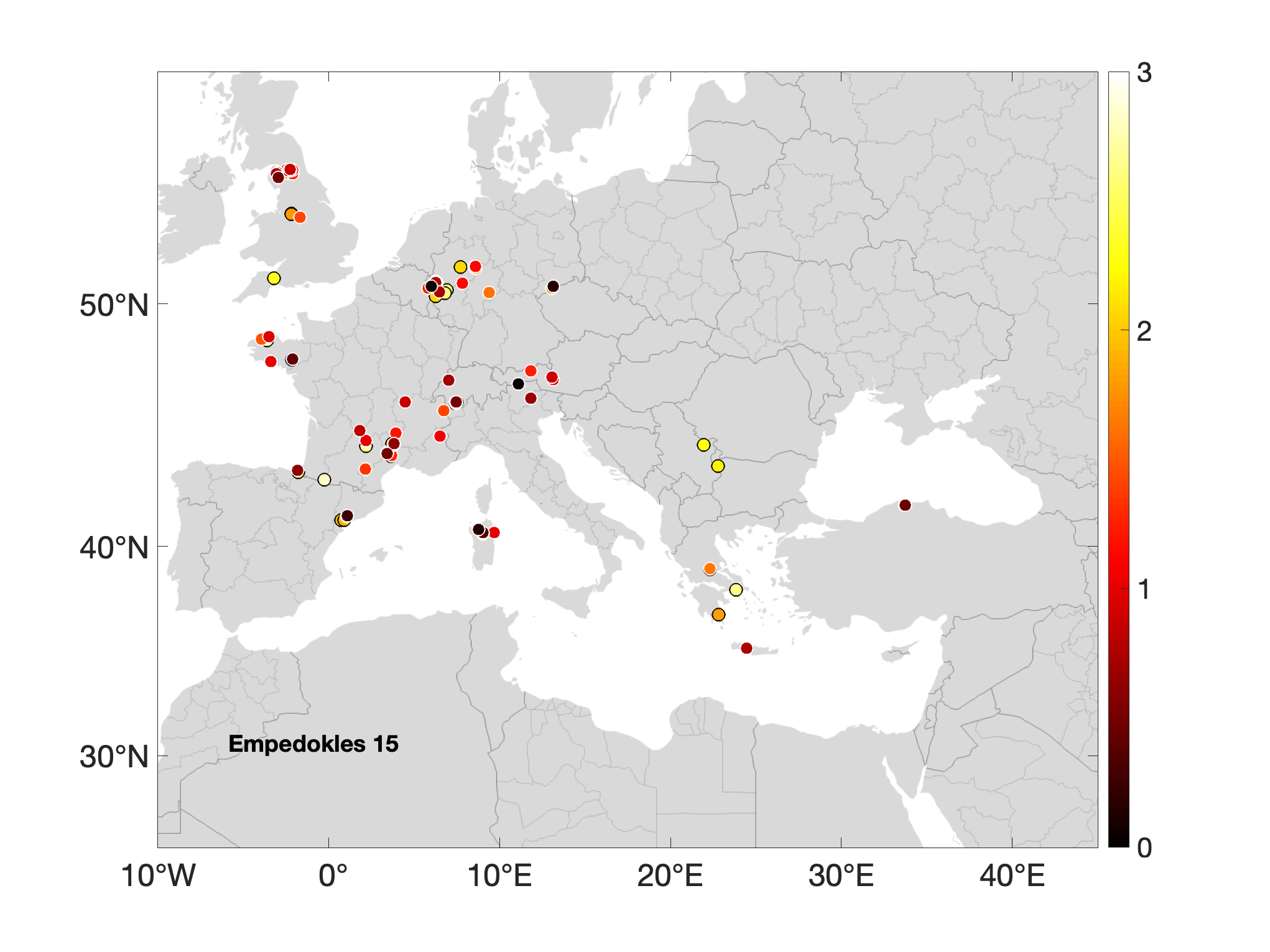

Supplement: Supplementary file 2 — Supplementary file2 (ZIP 8.20 MB) [file 12520_2024_2120_MOESM2_ESM.zip › png/Empedokles 15_map_jittered.png]

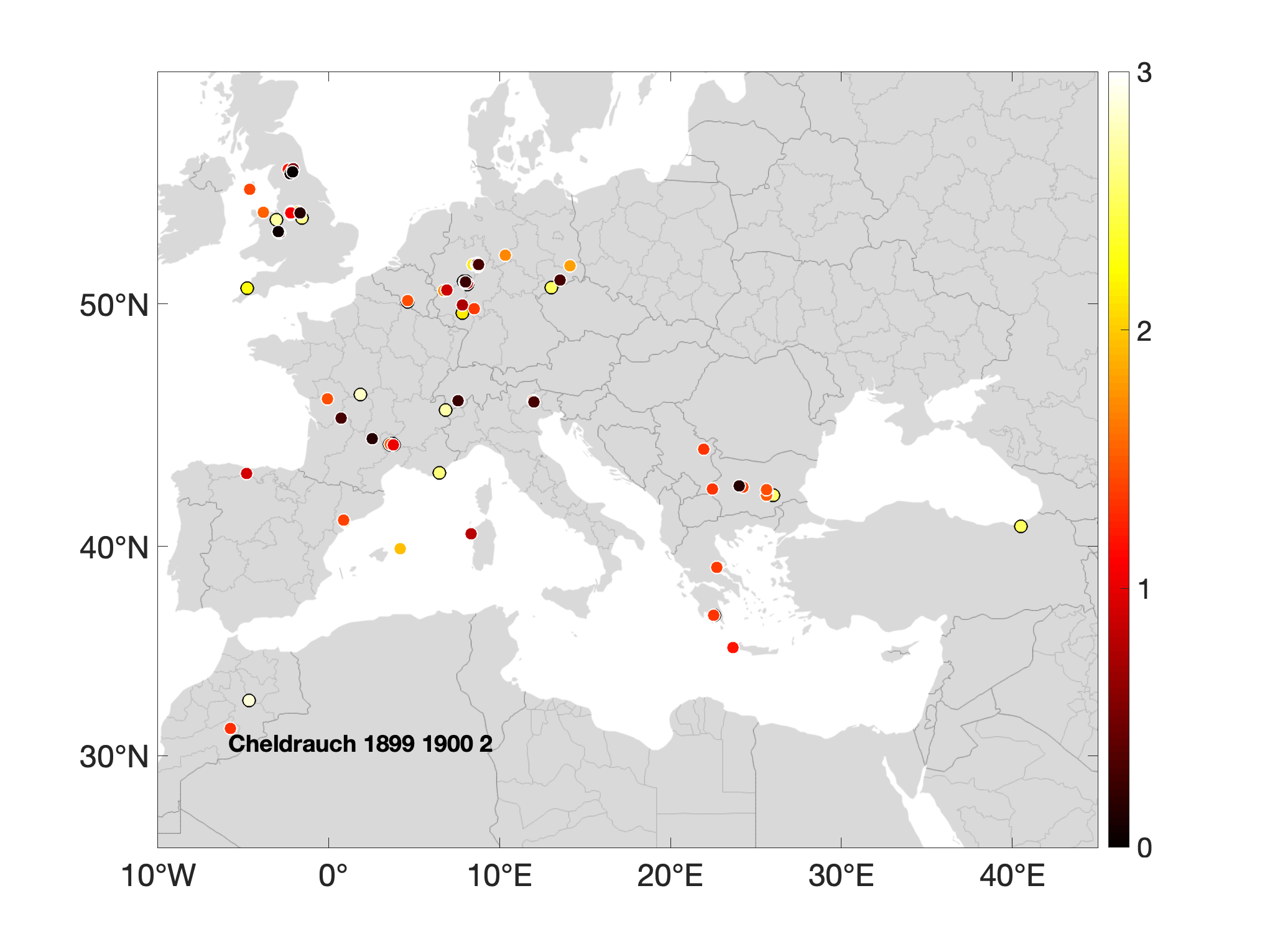

Supplement: Supplementary file 2 — Supplementary file2 (ZIP 8.20 MB) [file 12520_2024_2120_MOESM2_ESM.zip › png/Cheldrauch 1899 1900 2_map_jittered.png]

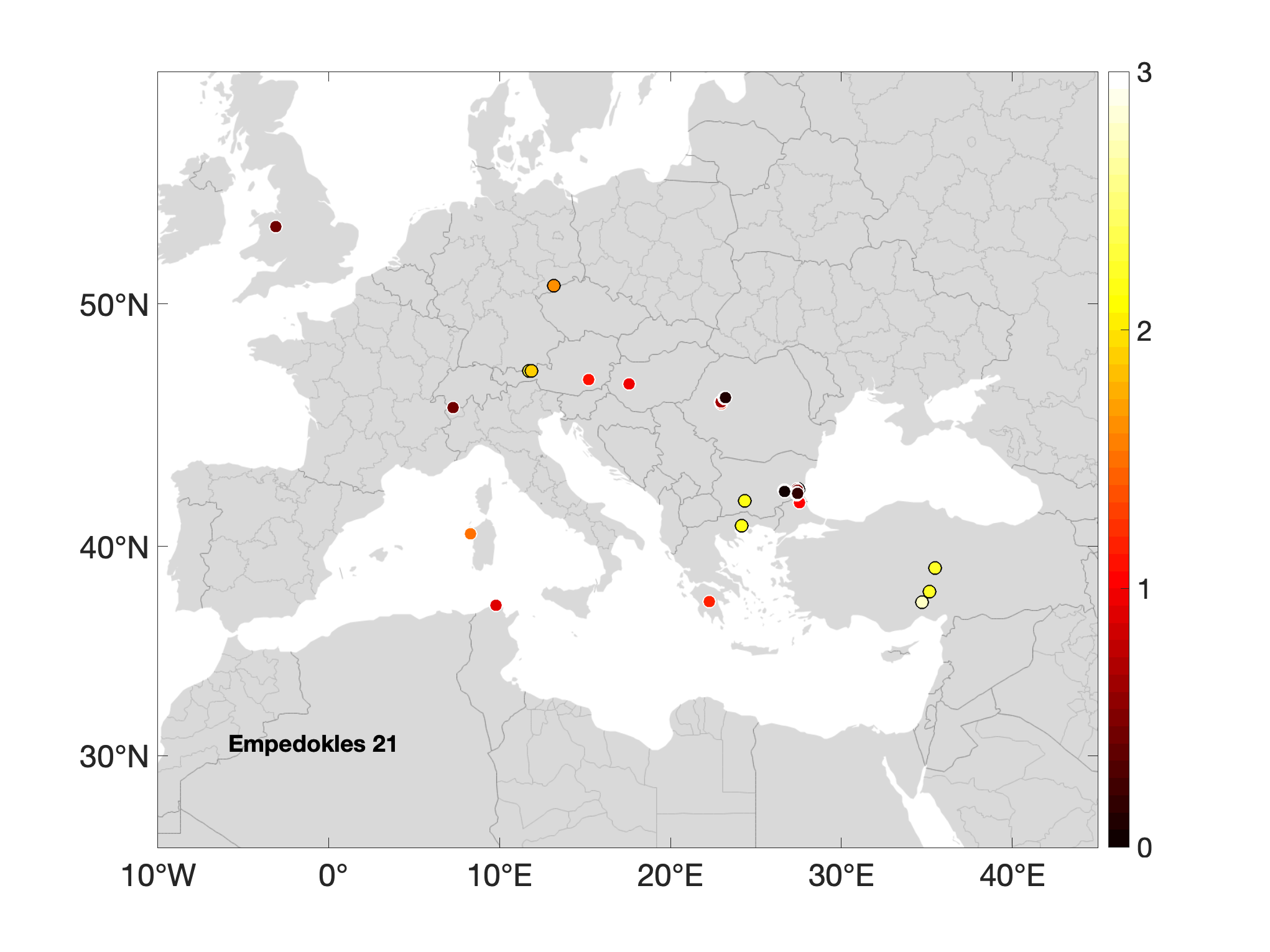

Supplement: Supplementary file 2 — Supplementary file2 (ZIP 8.20 MB) [file 12520_2024_2120_MOESM2_ESM.zip › png/Empedokles 21_map_jittered.png]

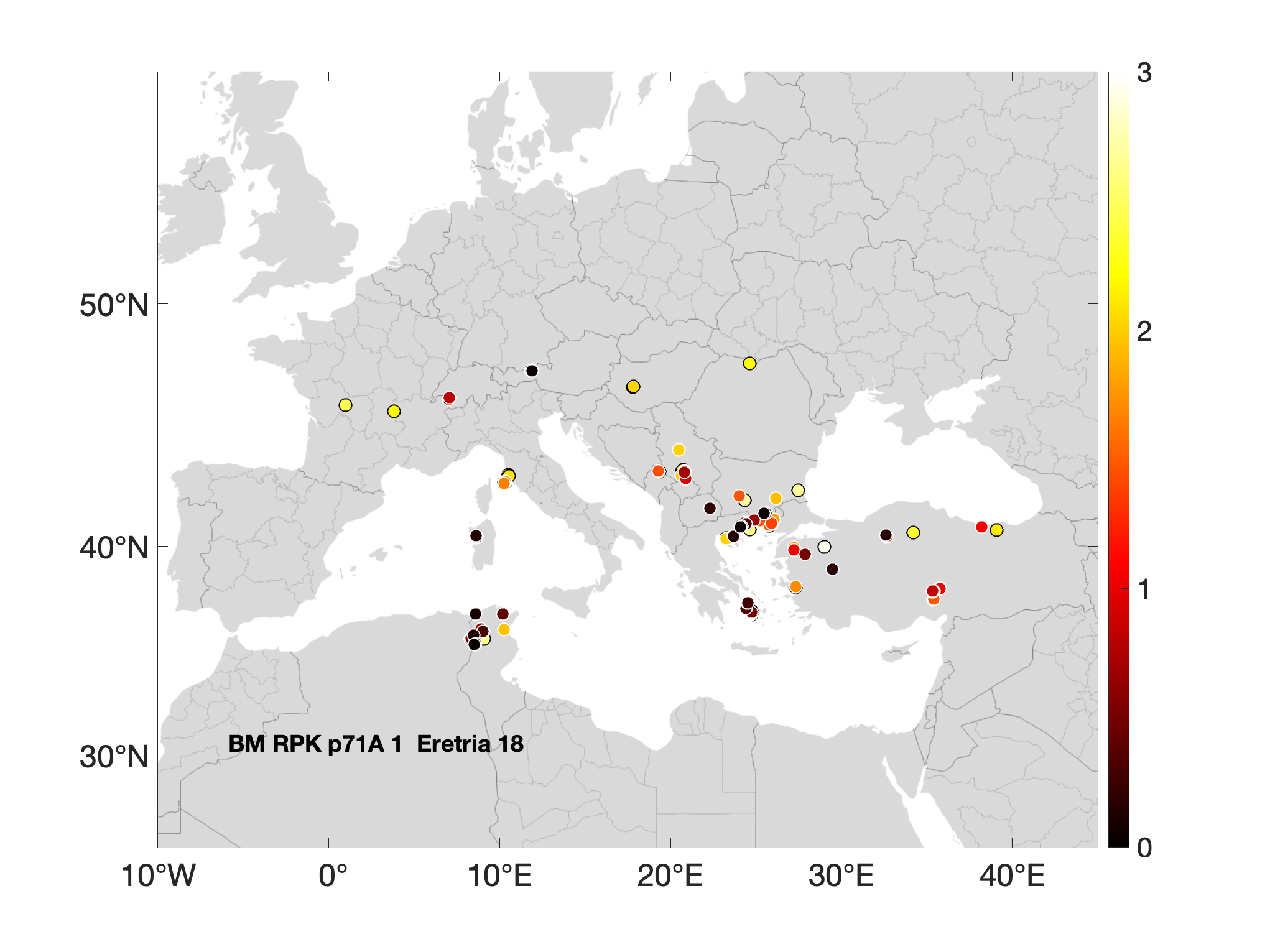

Supplement: Supplementary file 2 — Supplementary file2 (ZIP 8.20 MB) [file 12520_2024_2120_MOESM2_ESM.zip › png/BM RPK p71A 1 Eretria 18_map_jittered.png]

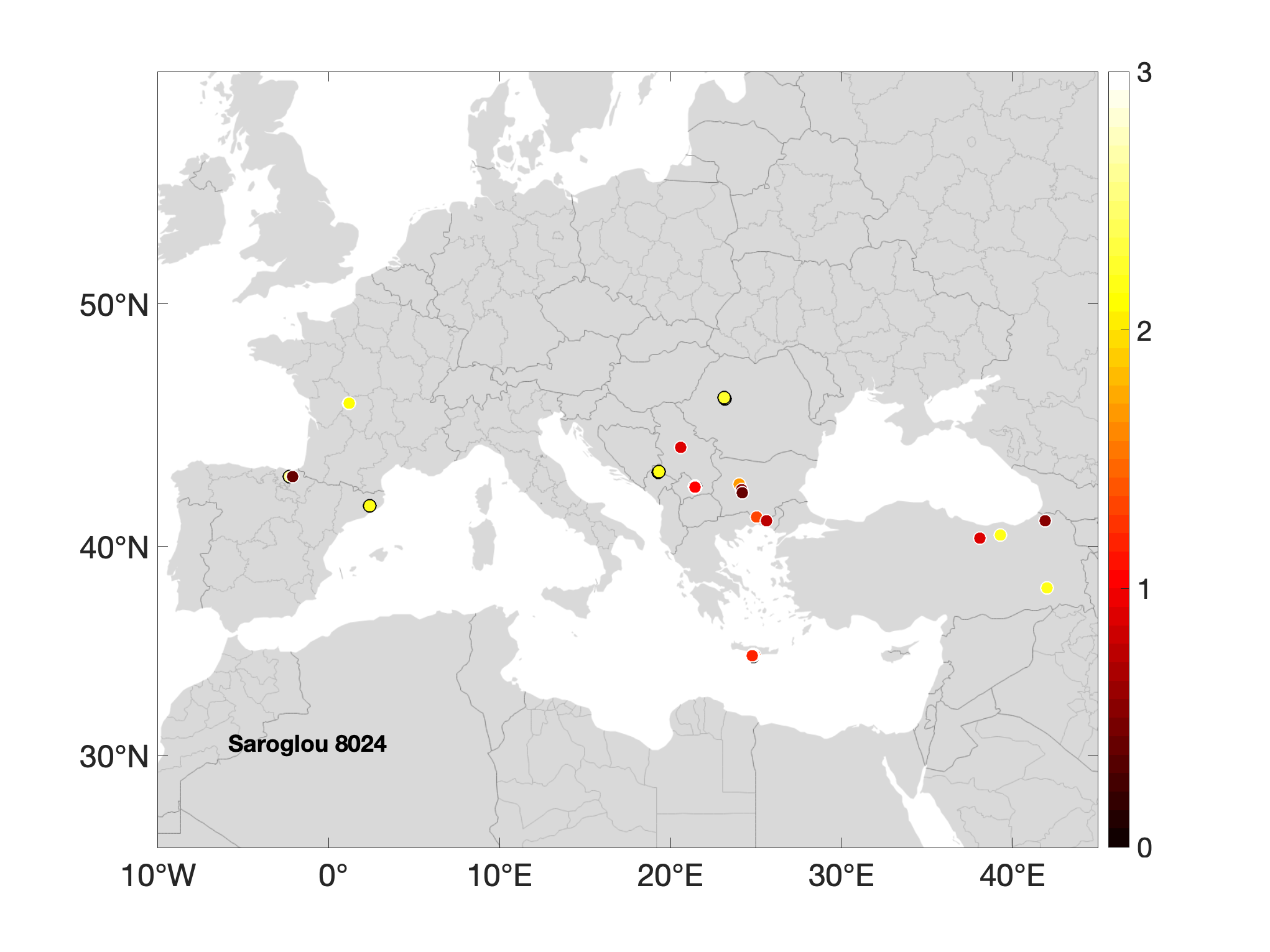

Supplement: Supplementary file 2 — Supplementary file2 (ZIP 8.20 MB) [file 12520_2024_2120_MOESM2_ESM.zip › png/Saroglou 8024_map_jittered.png]

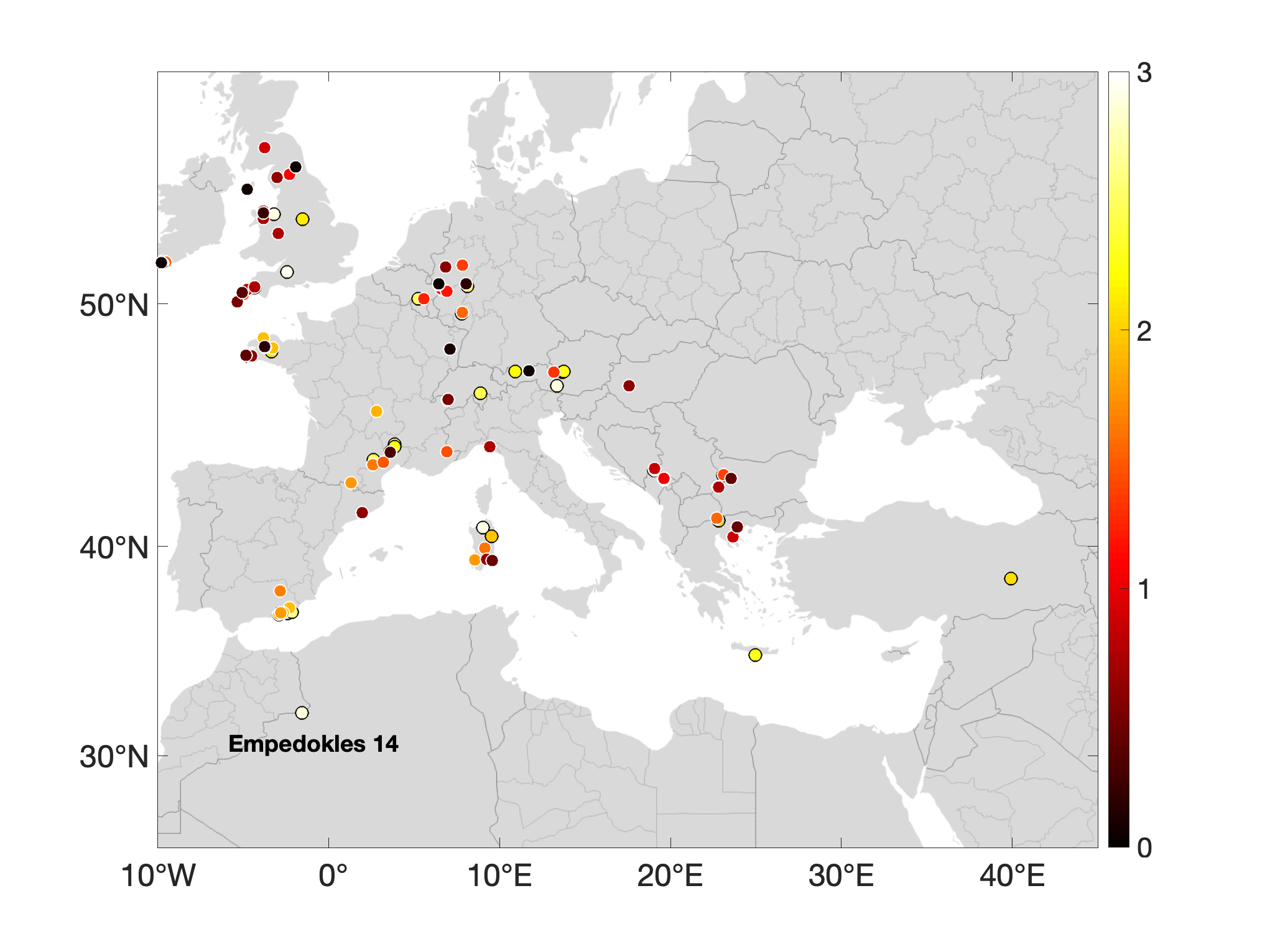

Supplement: Supplementary file 2 — Supplementary file2 (ZIP 8.20 MB) [file 12520_2024_2120_MOESM2_ESM.zip › png/Empedokles 14_map_jittered.png]

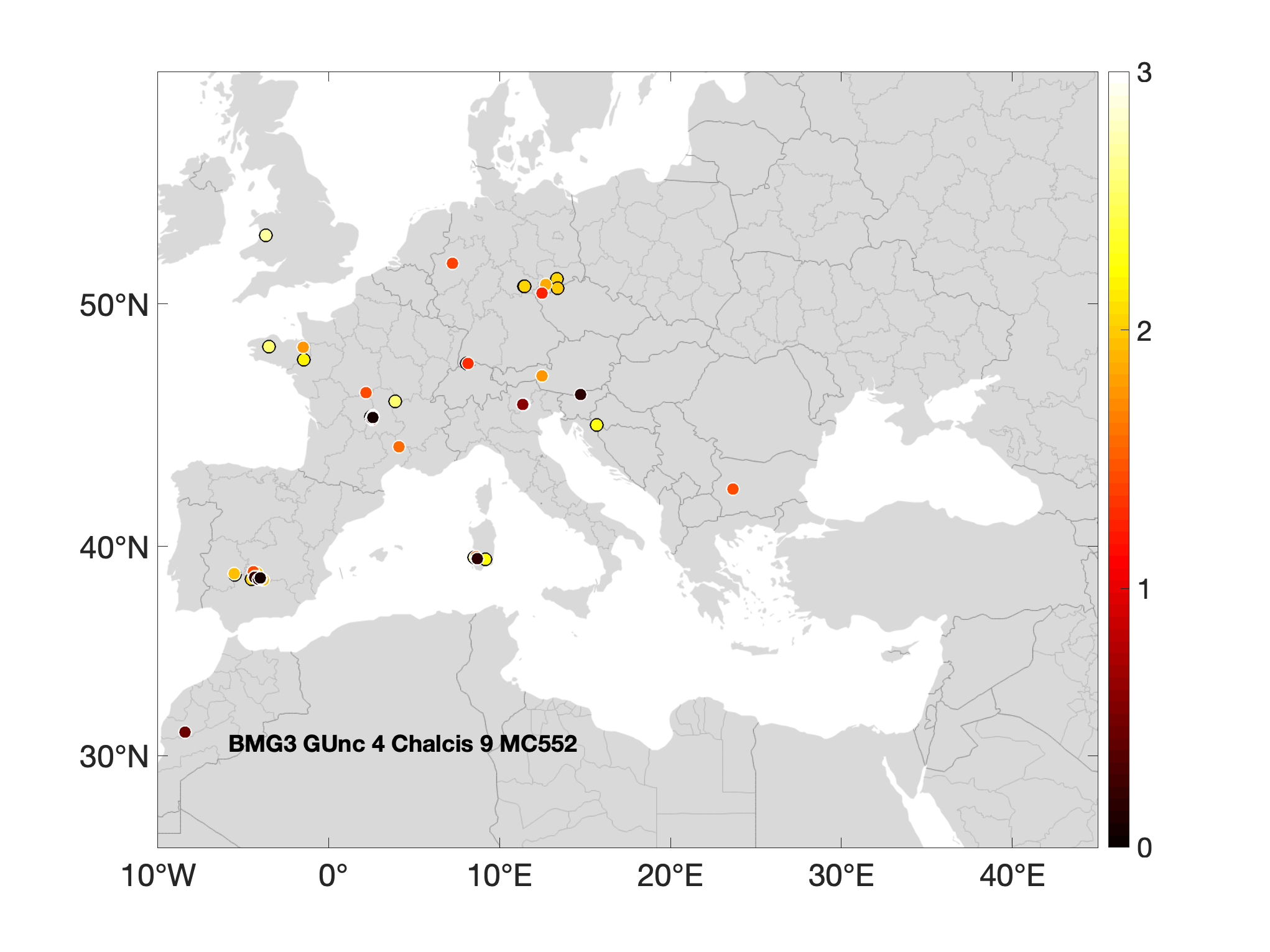

Supplement: Supplementary file 2 — Supplementary file2 (ZIP 8.20 MB) [file 12520_2024_2120_MOESM2_ESM.zip › png/BMG3 GUnc 4 Chalcis 9 MC552_map_jittered.png]

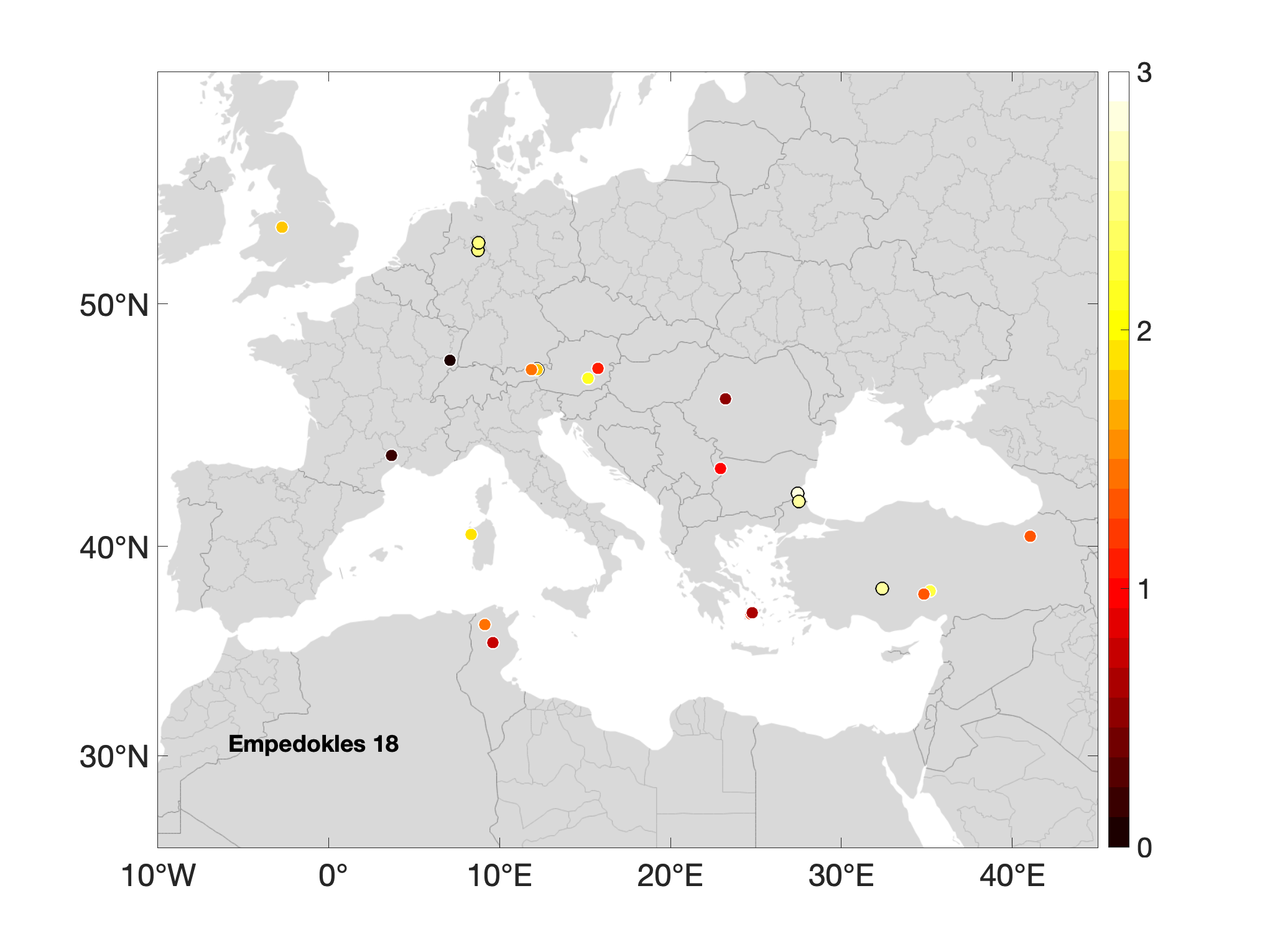

Supplement: Supplementary file 2 — Supplementary file2 (ZIP 8.20 MB) [file 12520_2024_2120_MOESM2_ESM.zip › png/Empedokles 18_map_jittered.png]

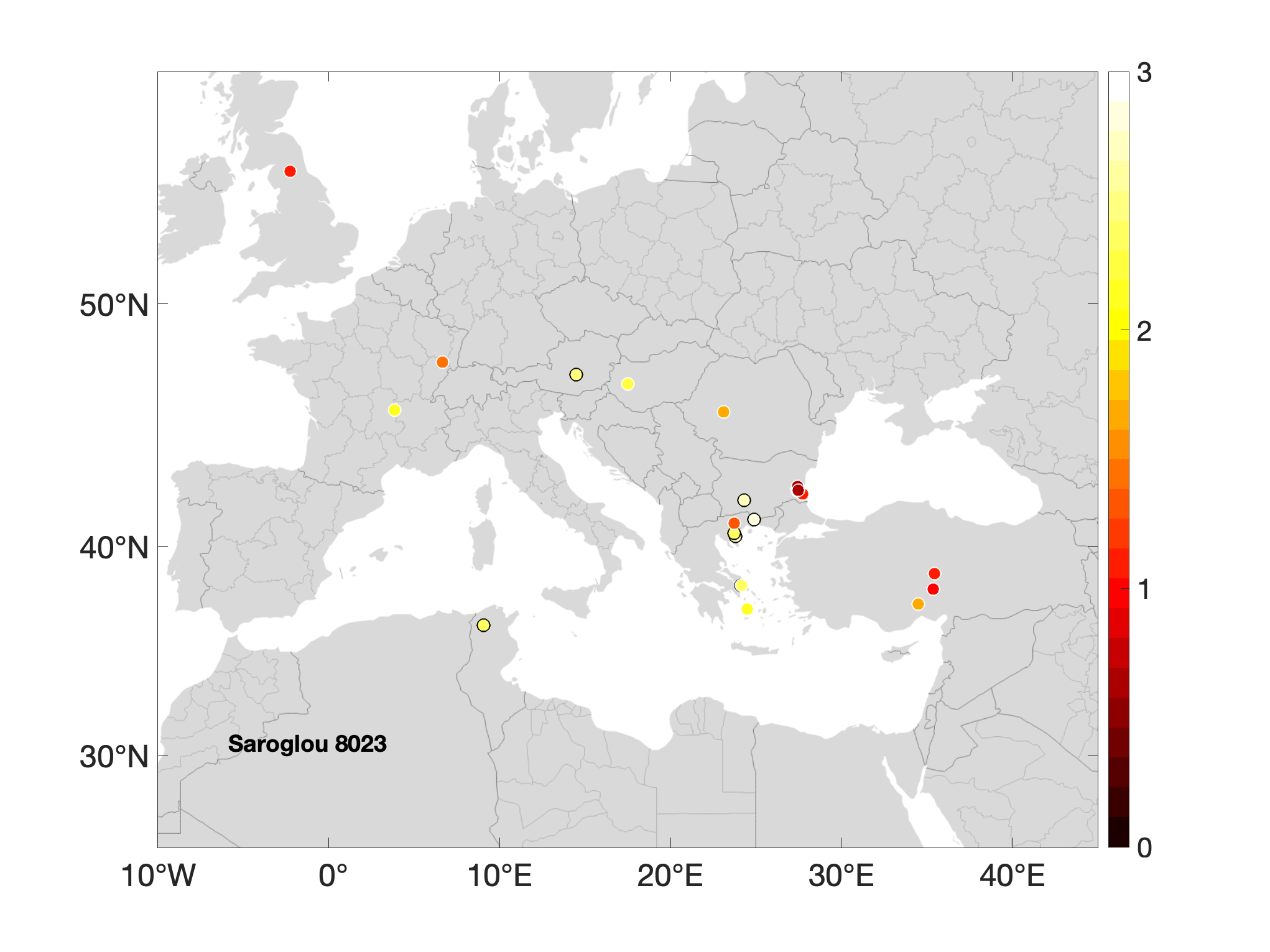

Supplement: Supplementary file 2 — Supplementary file2 (ZIP 8.20 MB) [file 12520_2024_2120_MOESM2_ESM.zip › png/Saroglou 8023_map_jittered.png]

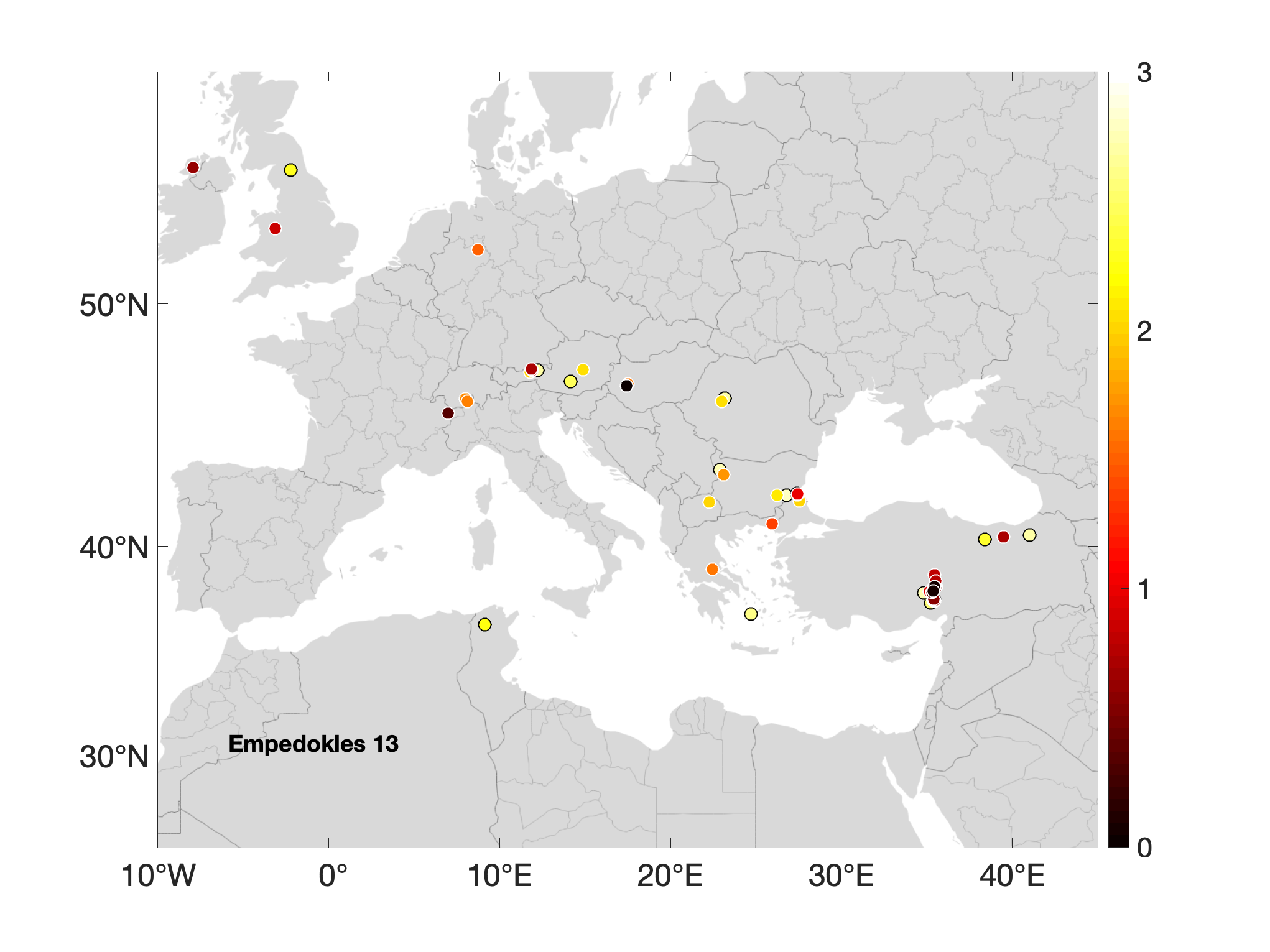

Supplement: Supplementary file 2 — Supplementary file2 (ZIP 8.20 MB) [file 12520_2024_2120_MOESM2_ESM.zip › png/Empedokles 13_map_jittered.png]

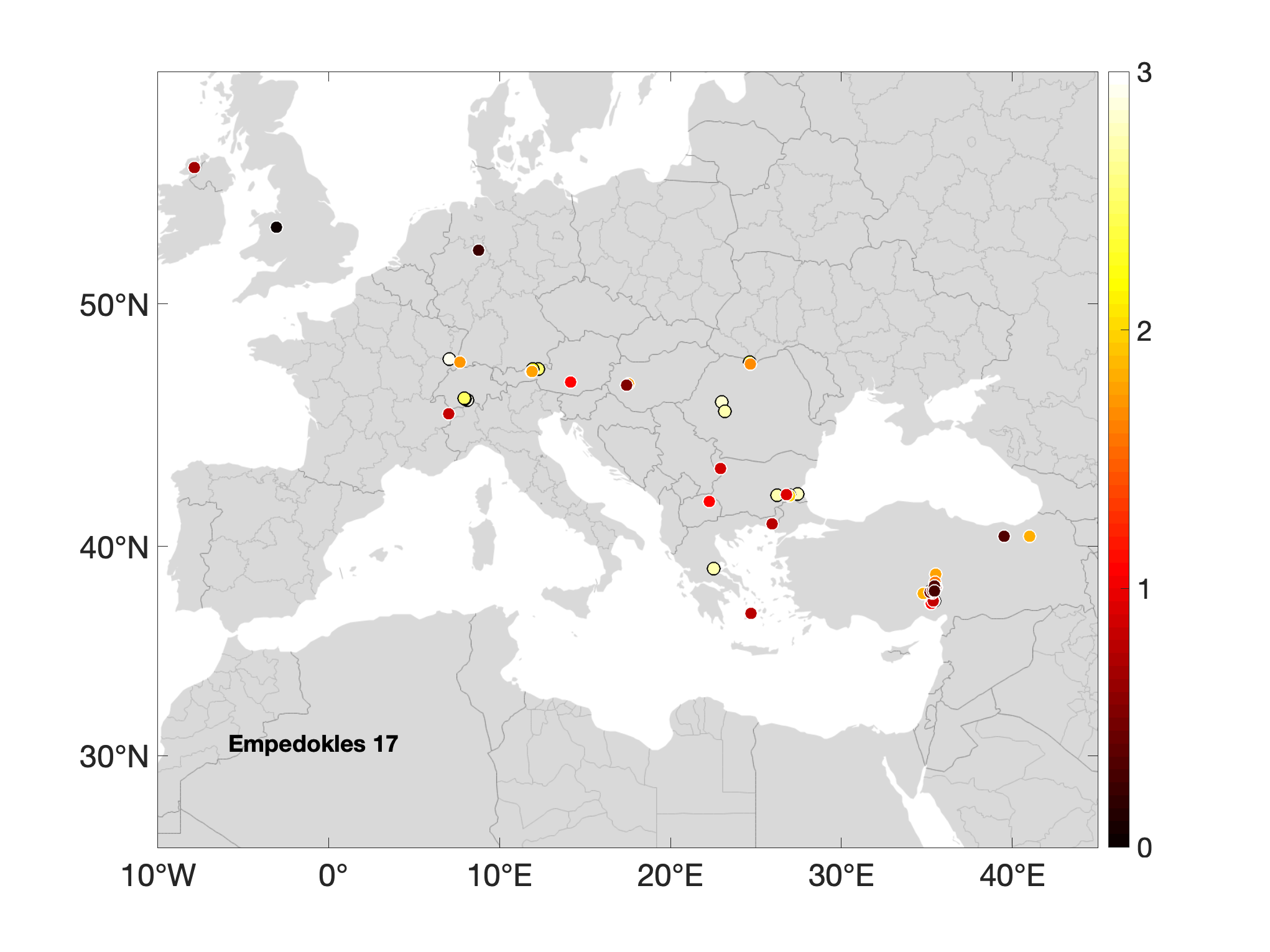

Supplement: Supplementary file 2 — Supplementary file2 (ZIP 8.20 MB) [file 12520_2024_2120_MOESM2_ESM.zip › png/Empedokles 17_map_jittered.png]

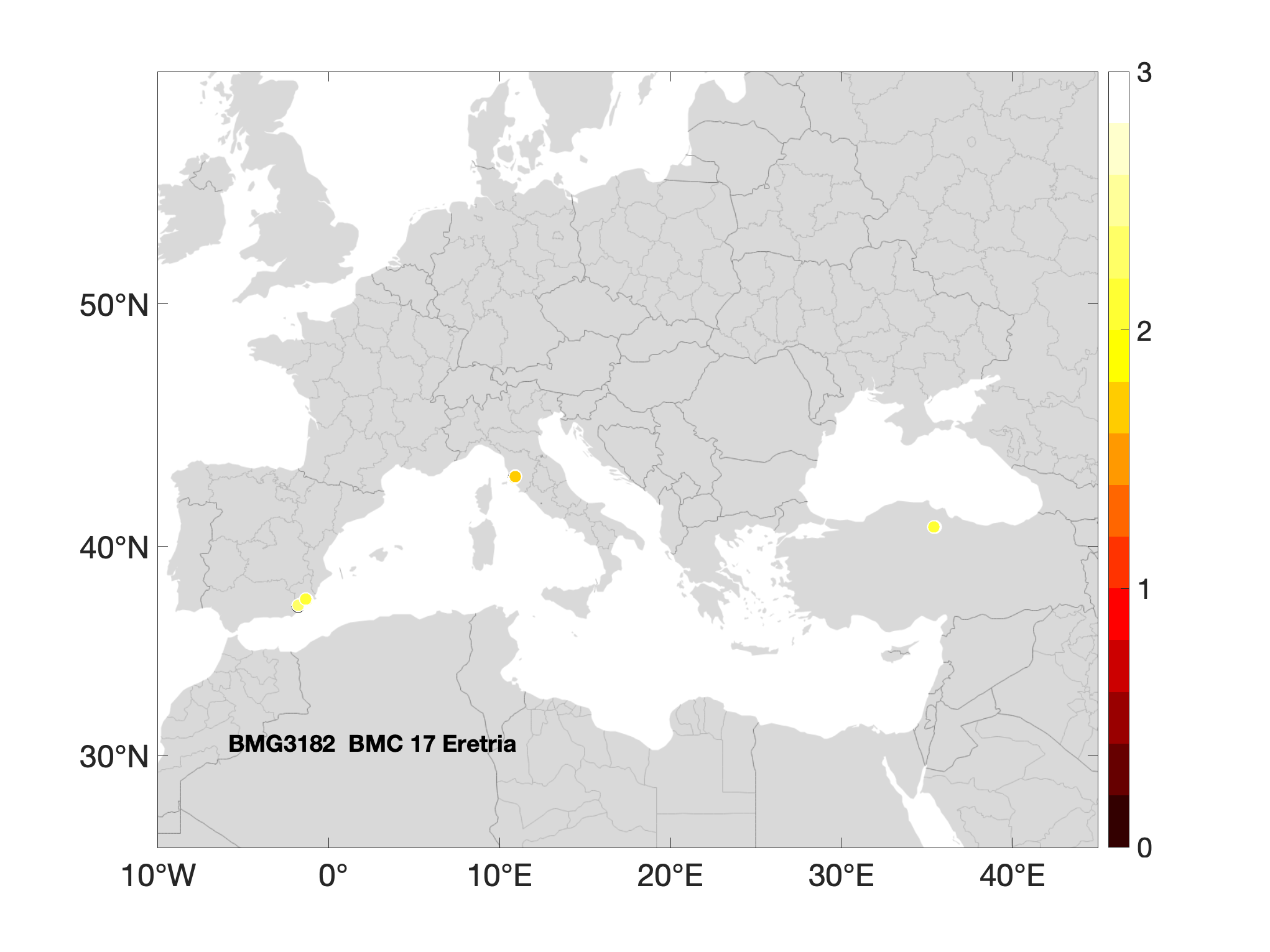

Supplement: Supplementary file 2 — Supplementary file2 (ZIP 8.20 MB) [file 12520_2024_2120_MOESM2_ESM.zip › png/BMG3182 BMC 17 Eretria_map_jittered.png]

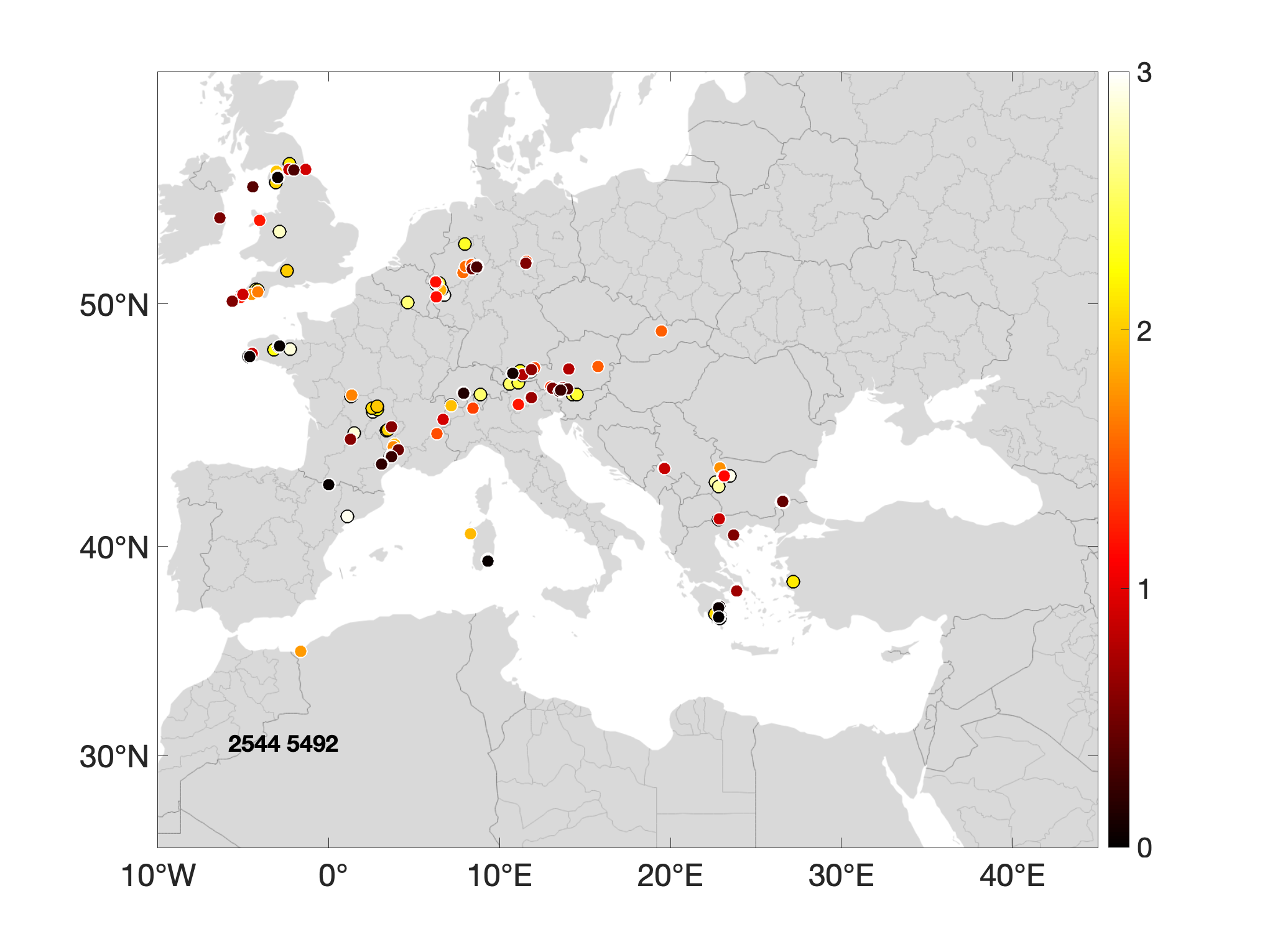

Supplement: Supplementary file 2 — Supplementary file2 (ZIP 8.20 MB) [file 12520_2024_2120_MOESM2_ESM.zip › png/2544 5492_map_jittered.png]

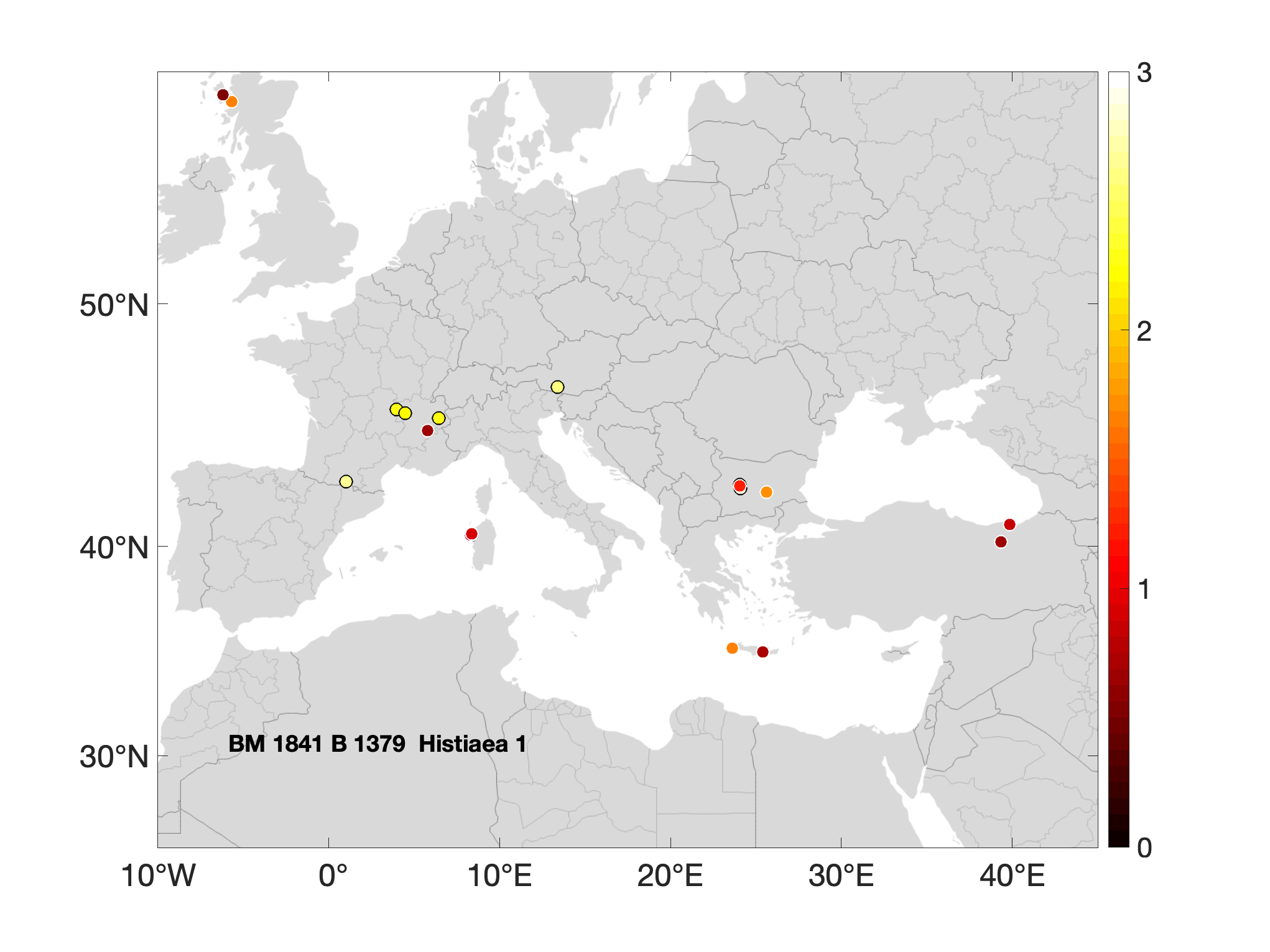

Supplement: Supplementary file 2 — Supplementary file2 (ZIP 8.20 MB) [file 12520_2024_2120_MOESM2_ESM.zip › png/BM 1841 B 1379 Histiaea 1_map_jittered.png]

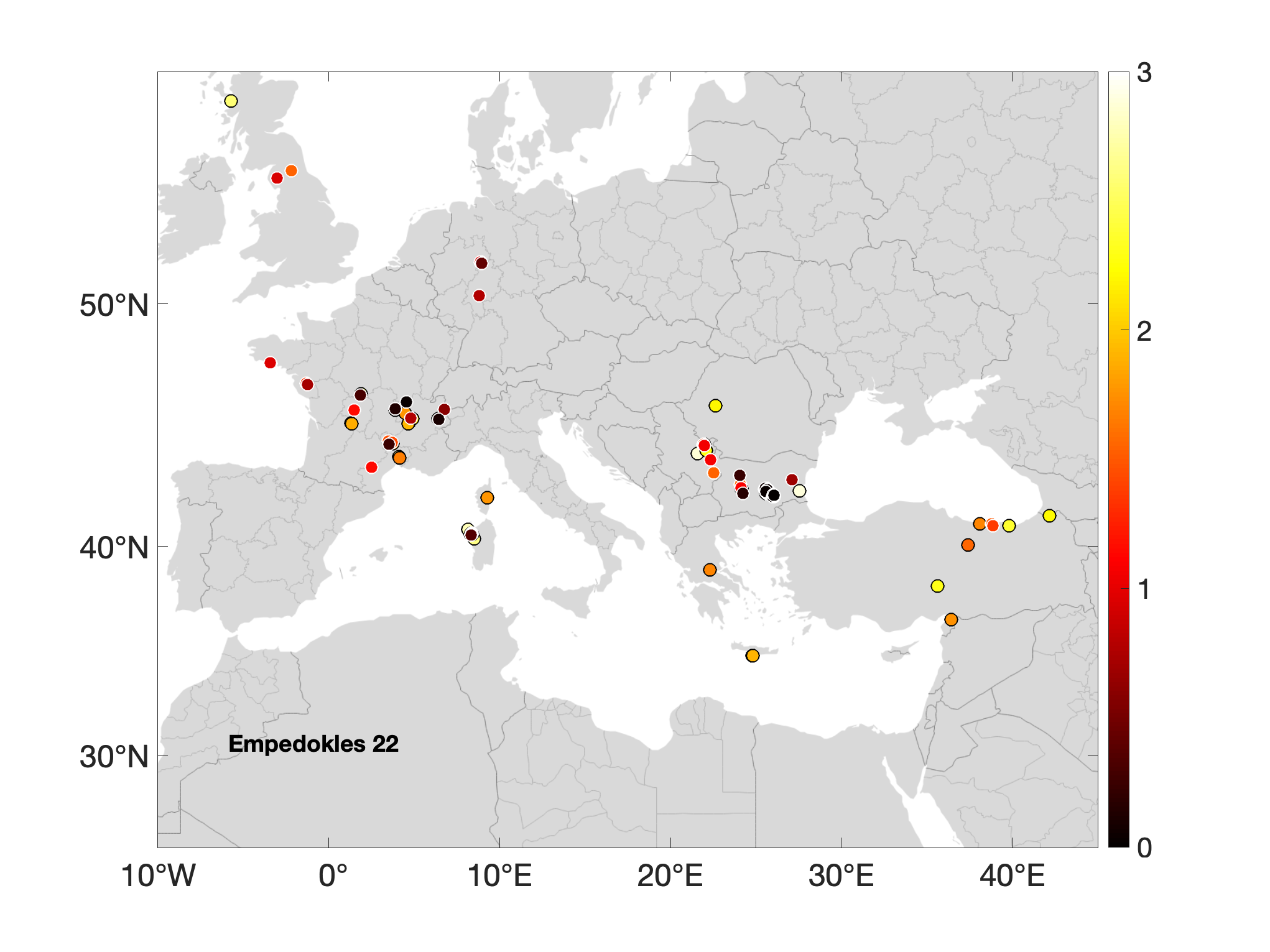

Supplement: Supplementary file 2 — Supplementary file2 (ZIP 8.20 MB) [file 12520_2024_2120_MOESM2_ESM.zip › png/Empedokles 22_map_jittered.png]

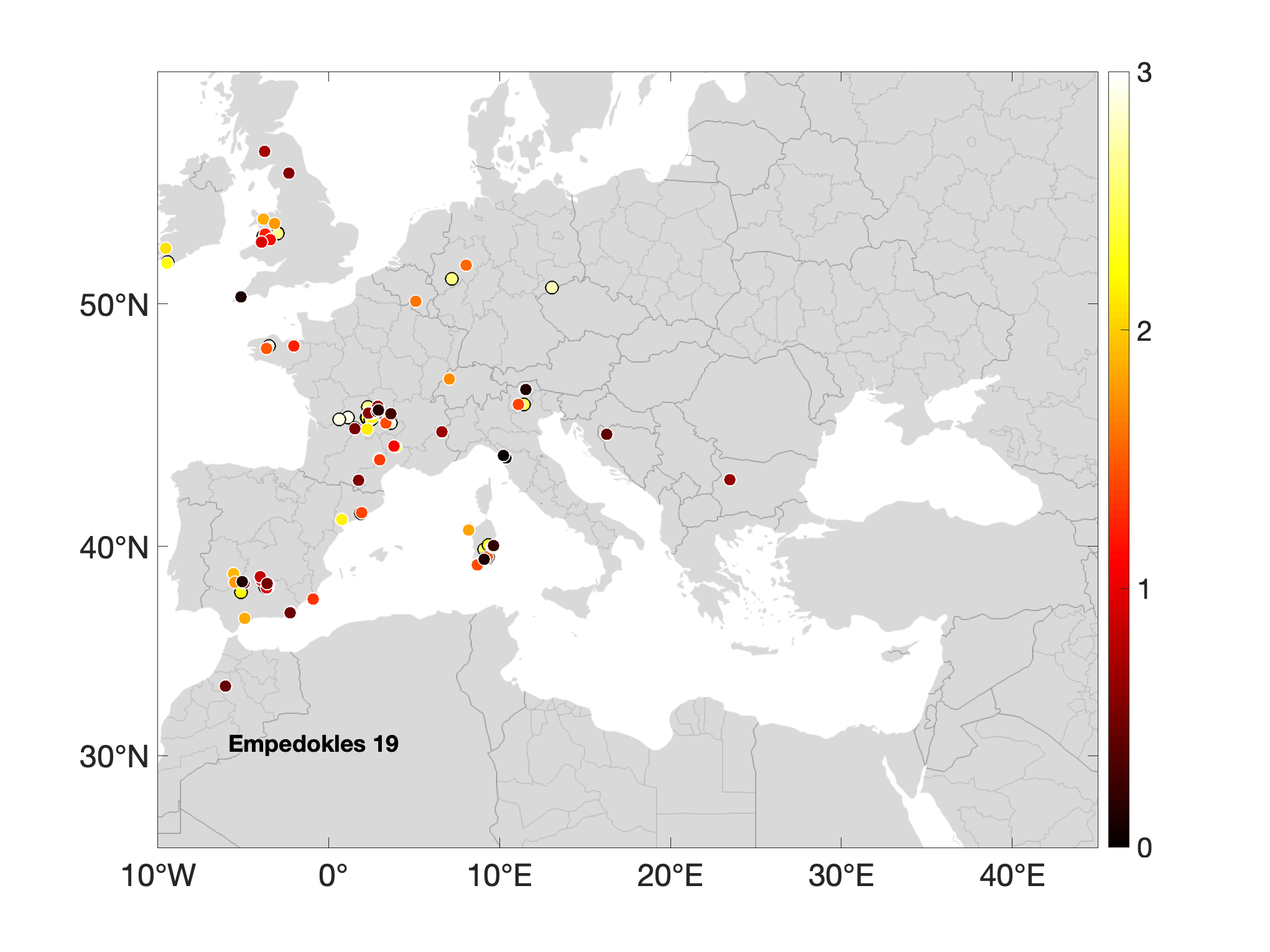

Supplement: Supplementary file 2 — Supplementary file2 (ZIP 8.20 MB) [file 12520_2024_2120_MOESM2_ESM.zip › png/Empedokles 19_map_jittered.png]

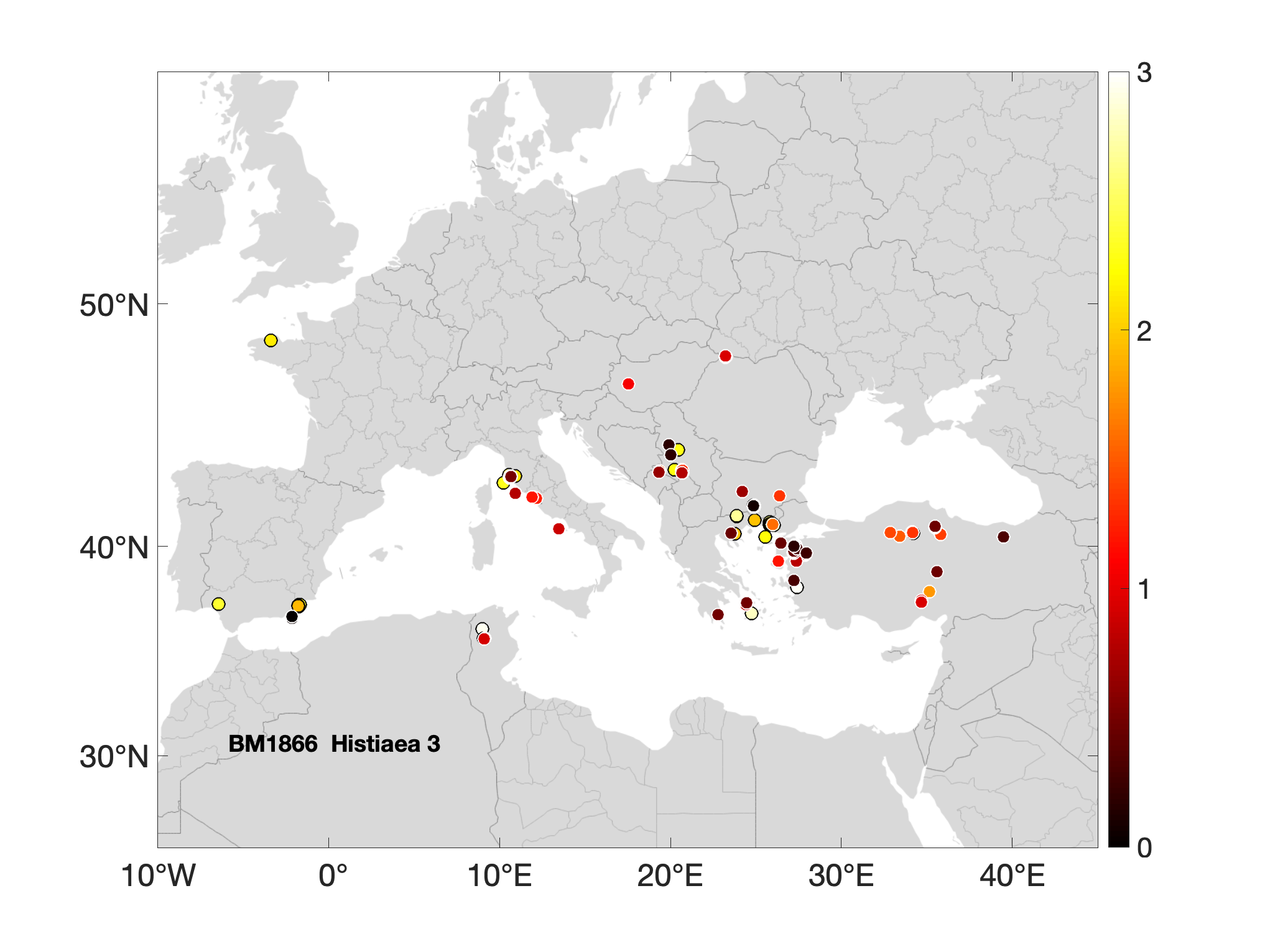

Supplement: Supplementary file 2 — Supplementary file2 (ZIP 8.20 MB) [file 12520_2024_2120_MOESM2_ESM.zip › png/BM1866 Histiaea 3_map_jittered.png]

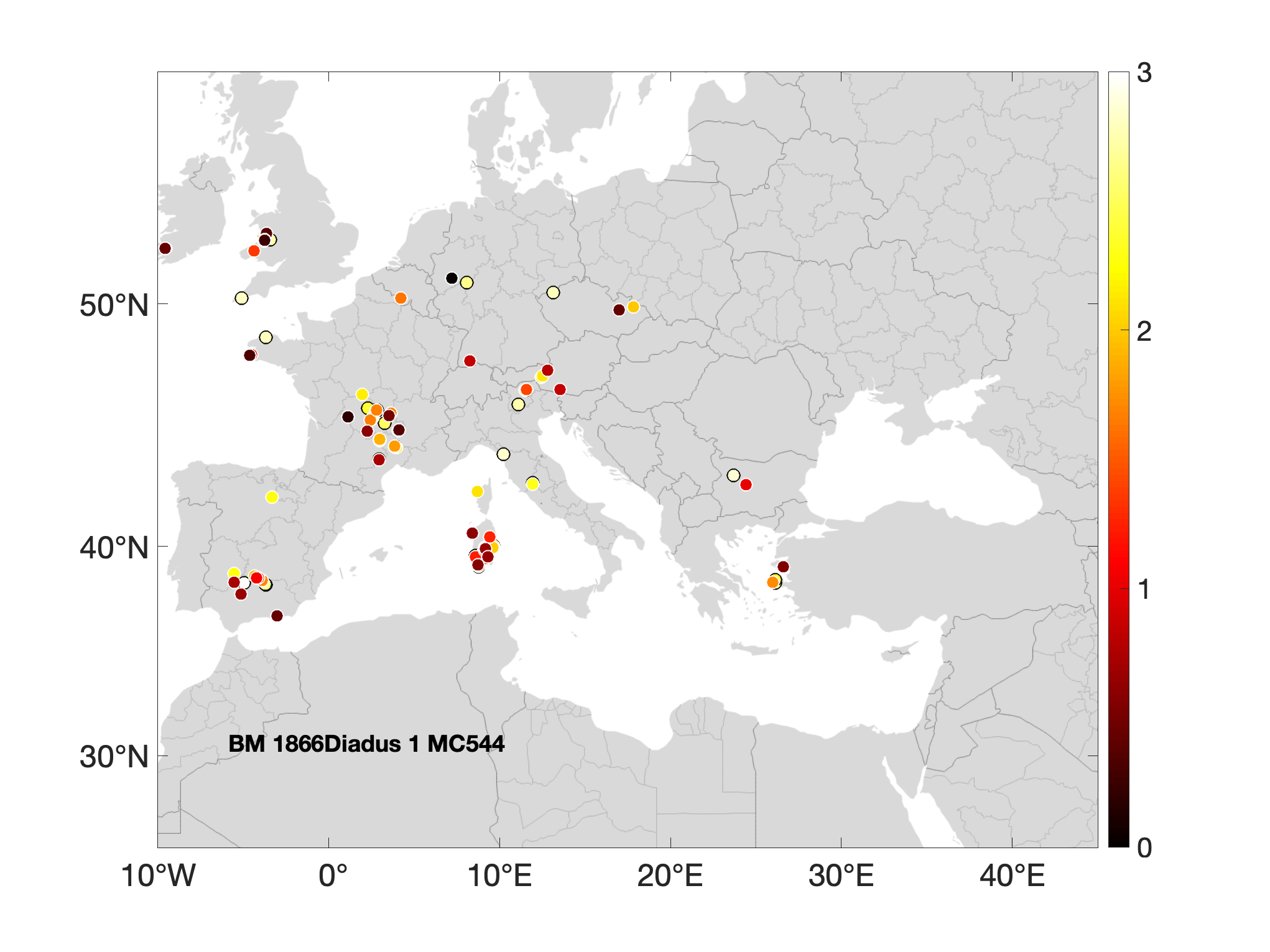

Supplement: Supplementary file 2 — Supplementary file2 (ZIP 8.20 MB) [file 12520_2024_2120_MOESM2_ESM.zip › png/BM 1866Diadus 1 MC544_map_jittered.png]

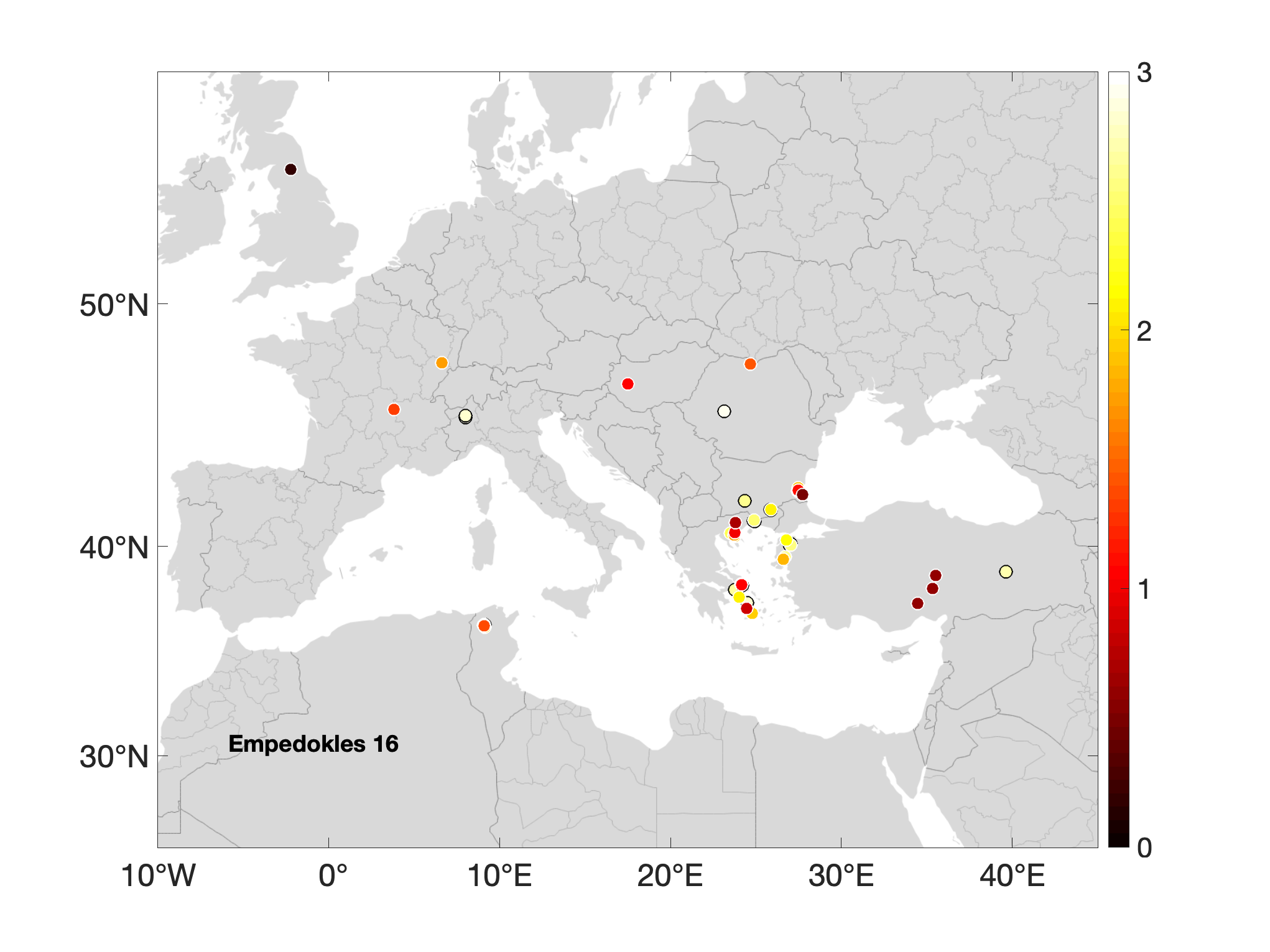

Supplement: Supplementary file 2 — Supplementary file2 (ZIP 8.20 MB) [file 12520_2024_2120_MOESM2_ESM.zip › png/Empedokles 16_map_jittered.png]

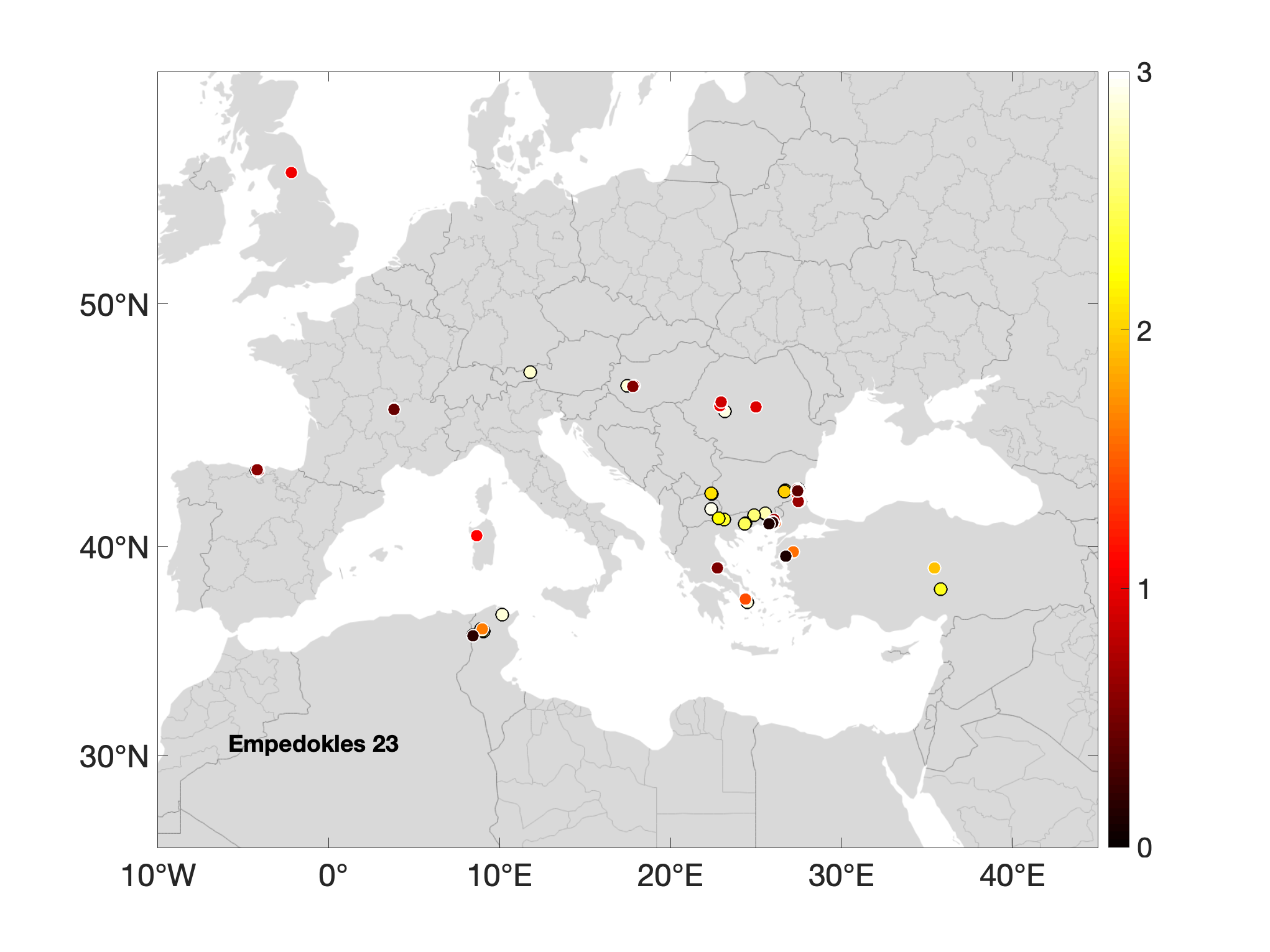

Supplement: Supplementary file 2 — Supplementary file2 (ZIP 8.20 MB) [file 12520_2024_2120_MOESM2_ESM.zip › png/Empedokles 23_map_jittered.png]
